# Enhanced nuclear translation is associated with proliferation and progression across multiple cancers

**DOI:** 10.1002/mco2.248

**Published:** 2023-04-14

**Authors:** Sailan Zou, Byung‐Wook Kim, Yan Tian, Geng Liu, Jiawei Zhang, Ricardo Zerda, Zhuo Li, Guixiang Zhang, Xiao Du, Weiqiang Lin, Xiang Gao, Wendong Huang, Xianghui Fu

**Affiliations:** ^1^ Division of Endocrinology and Metabolism State Key Laboratory of Biotherapy and Cancer Center West China Hospital Sichuan University and Collaborative Innovation Center of Biotherapy Chengdu China; ^2^ Department of Diabetes Complications and Metabolism Arthur Riggs Diabetes and Metabolism Research Institute Irell & Manella Graduate School of Biological Sciences Beckman Research Institute City of Hope National Medical Center Duarte USA; ^3^ Cancer Institute (Key Laboratory of Cancer Prevention and Intervention China National Ministry of Education) Second Affiliated Hospital School of Medicine Zhejiang University Hangzhou China; ^4^ Electron Microscopy and Atomic Force Microscopy Core City of Hope National Medical Center Duarte USA; ^5^ Division of Gastrointestinal Surgery Department of General Surgery and Gastric Cancer Center West China Hospital Sichuan University Chengdu China; ^6^ Department of General Surgery Yaan People's Hospital Yaan China; ^7^ Department of Nephrology The Fourth Affiliated Hospital International Institutes of Medicine School of Medicine Zhejiang University Zhejiang China; ^8^ Department of Neurosurgery and Institute of Neurosurgery State Key Laboratory of Biotherapy and Cancer Center West China Hospital West China Medical School Sichuan University and Collaborative Innovation Center for Biotherapy Chengdu China

**Keywords:** nucleolus, proliferation, proteomics, ribosome, translation, tumorigenesis

## Abstract

Recent technological advances have re‐invigorated the interest in nuclear translation (NT), but the underlying mechanisms and functional implications of NT remain unknown. Here we show that NT is enhanced in malignant cancer cells and is associated with rapid cell growth. Nuclear ribopuromycylation analyses in a panel of diverse cell lines revealed that NT is scarce in normal immortalized cells, but is ubiquitous and robust in malignant cancer cells. Moreover, NT occurs in the nucleolus and requires normal nucleolar function. Intriguingly, NT is reduced by cellular stresses and anti‐tumor agents and positively correlates with cancer cell proliferation and growth. By using a modified puromycin‐associated nascent chain proteomics, we further identified numerous oncoproteins that are preferentially translated in the nucleus, such as transforming growth factor‐beta 2 (TGFB2) and nucleophosmin 1 (NMP1). Specific overexpression of TGFB2 and NMP1 messenger RNAs in the nucleus can increase their protein levels and promote tumorigenesis. These findings establish a previously unknown link between NT and malignancy and suggest that cancer cells might have adapted a mechanism of NT to support their need for rapid growth, which highlight the potential of NT in tumorigenesis and might also open up new possibilities for therapeutic targeting of cancer‐specific cellular functions.

## Graphical abstract

Zou et al. reveal that nuclear translation (NT) is increased in cancer cells, leading to preferential production of oncogenic proteins and eventually tumorigenesis.

1) NT is dramatically enhanced in malignant cancer cells.

2) NT occurs in the nucleolus and relies on normal nucleolar function.

3) NT is markedly attenuated by cellular stresses and anti‐tumor agents.

4) Numerous oncoproteins are preferentially translated in the nucleus.

5) Nuclear‐specific delivery of an oncogenic messenger RNA can increase its protein levels and promote tumorigenesis.

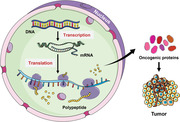

## 1 INTRODUCTION

A growing body of evidence suggests that translation can occur in the nucleus.^1^, ^2^, ^3^, ^4^, ^5^, ^6^, ^7^, ^8^, ^9^ Over a half‐century ago, Allfrey observed that highly purified calf thymus nuclei could incorporate radioisotope‐labeled amino acids into nuclear proteins and this incorporation requires intact DNA.^10^ Subsequent studies suggested that the nucleolus is a site of synthesis of nuclear proteins and some of these proteins are histones.^11^ Later, the presence of translation components, including various translation initiation/elongation factors, mature tRNAs, and ribosomes, was confirmed in the nucleus.^1^, ^12^, ^13^, ^14^, ^15^ Furthermore, messenger RNA (mRNA) surveillance mechanisms, such as nonsense‐mediated mRNA decay (NMD), have been observed in the nucleus.^16^, ^17^ NMD is initiated by ribosome recognition for premature termination codons in premature mRNAs, which provides evidence of active translation in the nucleus. Overall, these studies provide strong indications of nuclear translation (NT), albeit they have largely been ignored because of concerns about possible cytoplasmic contamination.^18^

Recent technological advances have further confirmed the existence of NT. By performing a series of careful experiments, David et al developed a new technique called ribopuromycylation (RPM) for imaging translation in intact cells. Intriguingly, RPM visualized robust synthesis of ribosome‐bound nascent peptide chains in the nucleolar compartment of HeLa cells and human monocytes, which was not caused by the import or trapping of nascent proteins from the cytoplasm.^2^ These results are consistent with a previous observation that pea nucleoli can incorporate radiolabeled amino acids into new proteins.^19^ In addition, Al‐Jubran et al. used bimolecular fluorescence complementation and showed that 80S ribosomes, a hallmark of translation initiation, were formed in the nucleoli of *Drosophila* S2 cells.^3^ More recently, by employing three approaches with different analogs (L‐azidohomoalanine, puromycin, and amino acids) and detection methods, Baboo et al. reported that most human proteins can be translated in both cytoplasm and nucleus and most newly‐synthesized peptides turn over within minutes.^20^

These recent observations narrow down the site of NT to the nucleolus, a prominent non‐membrane organelle within the eukaryotic nucleus. Conventionally, the nucleolus is known as the site of ribosome biogenesis, including rDNA transcription and processing, and assembly of the rRNAs and ribosomal proteins.^21^, ^22^, ^23^ However, accumulating evidence has implicated the nucleolus as a key regulator of various cellular processes that are central to maintaining normal cellular homeostasis.^24^ For example, the nucleolus functions as a “stress sensor”, which respond to a range of cellular stresses by initiating the tumor suppressor protein p53‐dependent or ‐independent nucleolar surveillance pathway. Consistent with the multifunctional nature of the nucleolus, its malfunction has been linked to various human diseases, such as Werner's syndrome, Treacher‐Collins syndrome, viral infections, and cancer.^25^, ^26^, ^27^ In particular, an increase in the number and size of nucleoli, common pathological features, and prognostic markers of malignancy, is closely associated with cancer development.^28^, ^29^ Despite a clear causal link between nucleolar malfunction and cancer, the underlying mechanisms remain poorly understood.

Here, we report a link between NT and cancer development. We performed nuclear RPM in various normal immortalized and cancer cell lines across multiple cancer types and showed that malignant cancer cells have active and robust NT in the nucleolus, which respond dynamically to cellular stresses and anti‐tumor agents. Furthermore, we profiled nuclear‐translated proteins by proteomic analyses and identified some cancerous proteins that are translated preferentially in the nucleus. Taken together, these findings establish a positive association between NT and cancer cell growth, providing evidence for the potential implications of NT in human diseases.

## 2 RESULTS

### 2.1 NT is enhanced in malignant cancer cells

RPM detects active ribosomal‐dependent protein translation using puromycin (PMY), a Tyr‐tRNA mimetic, to label nascent peptides.^2^ RPM analysis in A375 cells without PMY treatment showed no PMY staining (Figure S1), confirming the specificity of the antibody. Next, we used regular RPM and nuclear RPM (NRPM) to detect cytoplasmic translation (CT) and NT, respectively. Compared to the diffuse pattern of CT, NT was more condensed and restricted to a specific area in the nucleus (Figure S2). Intriguingly, PMY signals were much stronger in malignant cancer cell lines (PC3 and MB‐MDA‐231) than in normal immortalized human fibroblast cell lines (IMR90 and MRC5) (Figure S2), indicating an enhancement of NT in malignant cancer cells.

**FIGURE 1:**
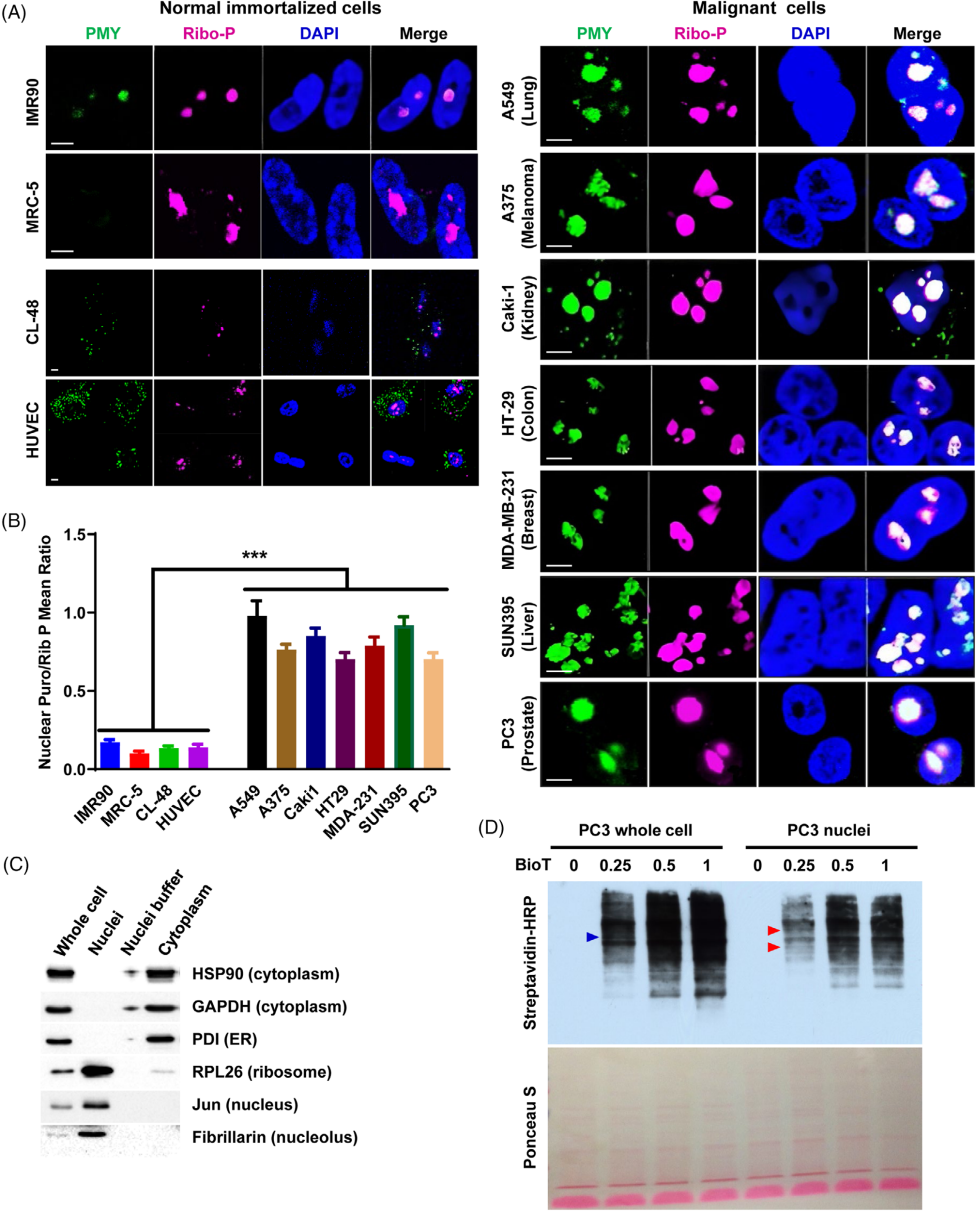
Enhanced nuclear translation in malignant cancer cells. (A, B) NRPM analysis on normal immortalized cells (left panel) and malignant cancer cells (right panel) across seven cancer types as indicated (A). Scale bar: 5 µm. Twenty fields were acquired for each condition, and the mean fluorescence ratio of PMY/Ribosome P staining for each field was quantitated using ImageJ (B). (C) The purity of isolated PC3 nuclei was determined by western blotting using the antibodies against specific marker proteins as indicated. (D) Newly synthesized proteins from whole cells or isolated nuclei of PC3 cells were labeled by biotinylated Puromycin (BioT) and detected by western blotting using streptavidin‐horseradish peroxidase (HRP). Triangles indicate potential distinct proteins. Ponceau S staining is shown as a control. Data are shown as mean ± SEM. ***p < 0.005. Two‐tailed unpaired *t*‐test. PMY: puromycin; Ribo‐P: ribosomal P.

To test this idea, we used NRPM to expand the analysis of NT in a panel of cell lines, which consists of 4 normal immortalized cell lines and 17 malignant cancer cell lines across seven cancer types, including breast, colon, kidney, liver, lung, skin, and prostate cancer (Figures 1A and Figure S3). Faint but clear PMY staining was detected in the nuclei of normal immortalized cells, particularly IMR90 (Figure 1A, left panel), indicating that normal immortalized cells preserve the ability for NT. Intriguingly, nuclear PMY staining was strong and intense in all malignant cancer cell lines that we examined (Figure 1A, right panel; and Figure S3). Quantitation of the mean fluorescence ratio of PMY/ribosome staining showed that NT is enhanced in malignant cancer cells (Figure 1B). In addition, high‐resolution confocal imaging showed considerable colocalization of nuclear PMY and ribosomal P in both normal immortalized and cancer cells (Figures 1A and Figure S3), consistent with the importance of ribosomes in protein translation.

In addition to NRPM, we adopted the strategy from the previously described method termed puromycin‐associated nascent chain proteomics (PUNCH‐P)^30^ to further confirm NT in malignant cancer cells, in which newly synthesized proteins from isolated nuclei were labeled with biotinylated PMY (BioT) and detected by western blotting (Figure S4A). The purity of the nuclei was confirmed by trypan blue staining (Figure S4B) and western blotting for cytoplasmic and nuclear markers (Figure 1C). In addition, to ascertain the amount of BioT required for complete labeling of newly synthesized peptides, we incubated a fixed amount of ribosomes with increasing amounts of BioT and found that a ratio of 0.5 pmol of BioT to 1 OD254 ribosomes was suitable for complete labeling (Figure 1D). This analysis revealed that PC3 nuclei indeed had active protein translation. Moreover, the repertoire of labeled proteins in PC3 nuclei seemed to be different from that of PC3 whole cell (Figure 1D), arguing against that protein translation detected in nuclei resulted from cytoplasmic contamination. These results collectively show that NT is scarce in normal immortalized cells, but is robust and intense in malignant cancer cells, indicating a potential association of NT with tumorigenesis.

### 2.2 NT occurs within the nucleolus

**FIGURE 2:**
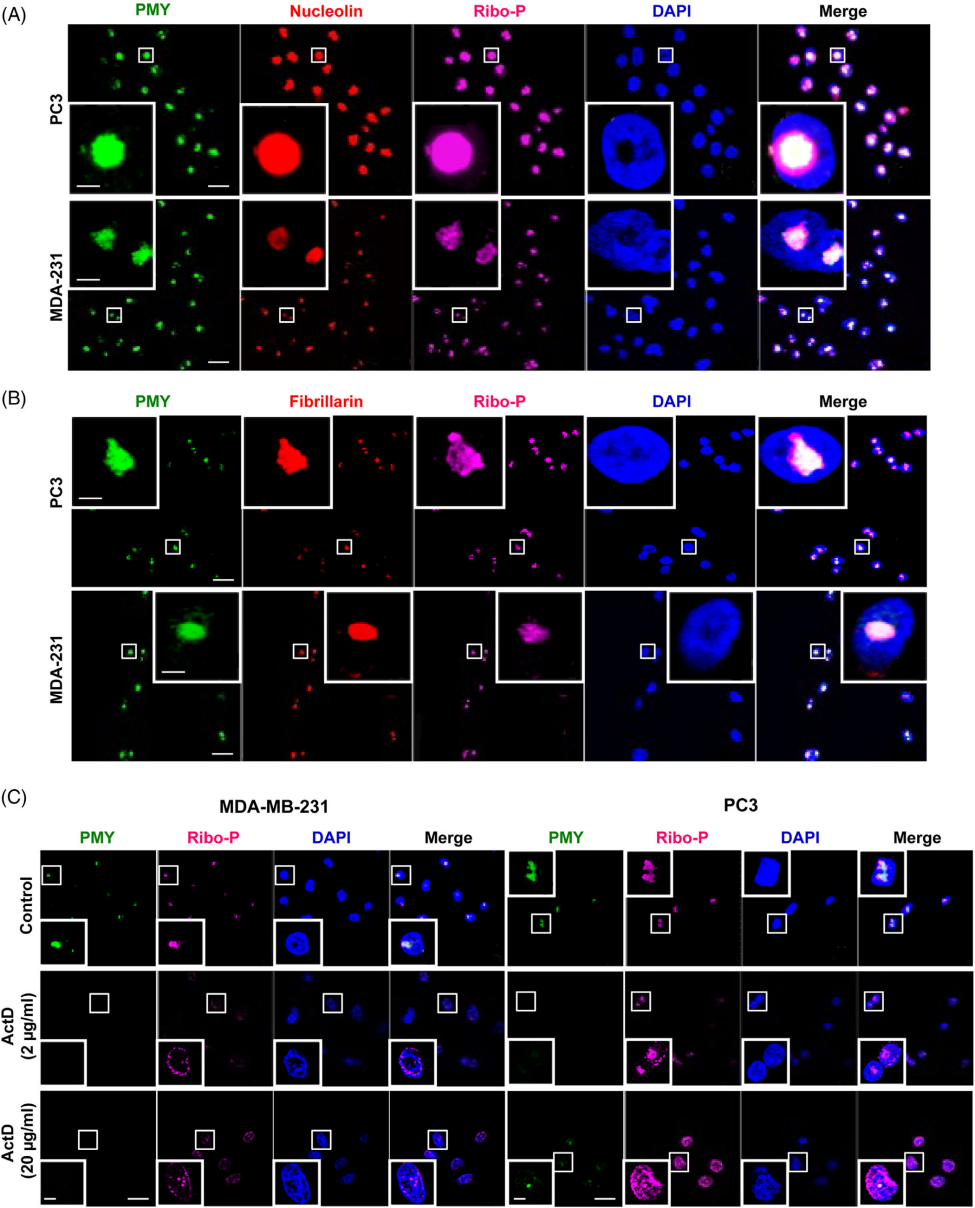
Nuclear translation in the nucleolus. (A, B) Co‐localization of nuclear translation and the nucleolar markers nucleolin (A) or fibrillarin (B) in PC3 and MDA‐MB‐231 cells. Scale bar: 20 µm; inserted magnified view: 5 µm. (C) MDA‐MB‐231 (left panel) and PC3 cells (right panel) were treated with actinomycin D (ActD) and the cells were then subjected to NRPM analysis. Scale bar: 20 µm; inserted magnified view: 5 µm. PMY: puromycin; Ribo‐P: ribosomal P.

Compared to CT, NT displayed a condensed and restricted pattern (Figure S2). Recent studies provide some evidence for the enrichment of functional, translational competent ribosomes in the nucleolus, which thus is implicated as the major site of NT.^3^ To verify this, we used co‐immunolocalization to determine the location of NT relative to the nucleolus. As expected, the PMY staining perfectly overlapped with the nucleolar marker nucleolin in normal immortalized cells and cancer cells that we examined, including IMR90, MRC5, PC3, and MDA‐MB‐231 (Figures 2A and Figure S2). Moreover, this colocalization also overlapped with ribosomal proteins, as shown by the ribosomal P antibody. Similarly, the colocalization of PMY and ribosomal P overlapped with fibrillarin (Figure 2B), another classic marker of the nucleolus. Of note, normal immortalized cells (IMR90 and MRC5) displayed similar intensity of nucleolin and ribosomal P as MDA‐MB‐231 cells, but their nuclear PMY staining was much weaker than that of MDA‐MB‐231 cells (Figure S2), suggesting that certain undetermined factors, in addition to nucleolin and ribosomes, are required for active NT.

We further determined if NT depends on the normal nucleolar function. To this end, we treated cells with actinomycin D (ActD), an inhibitor of rRNA synthesis that impairs nucleolar function,^31^ prior to NRPM analysis. The treatment of ActD led to the diffuse pattern of ribosomal proteins (Figure 2C), indicating the loss of normal nucleolar structure and function. Importantly, this treatment nearly abolished nucleolar RPM staining in both MDA‐MB‐231 and PC3 cells (Figure 2C). Taken together, these results strongly suggest that NT occurs at the nucleolus and requires normal nucleolar function, albeit the latter may be insufficient to initiate/maintain NT.

### 2.3 NT is reduced by cellular stresses and anti‐tumor agents

**FIGURE 3:**
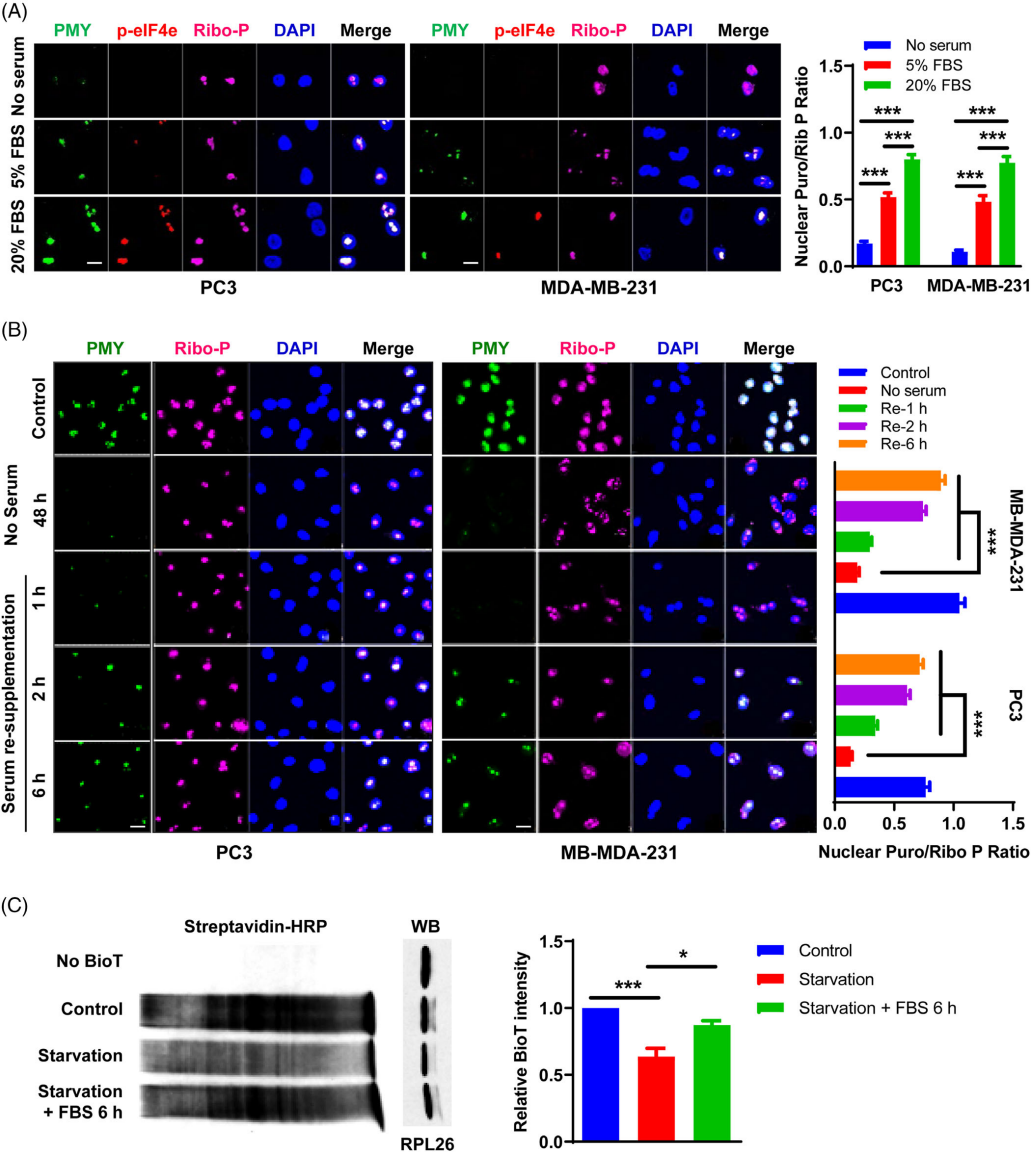
Reduced nuclear translation in malignant cancer cells during serum starvation. (A) PC3 (left panel) and MDA‐MB‐231 (middle panel) cells were cultured with different concentrations of fetal bovine serum (FBS) as indicated, followed by the NRPM analysis. Scale bar: 10 µm. Fifteen fields were acquired for each condition, and the mean fluorescence ratio of PMY/Ribosome P staining for each field was quantitated using ImageJ (right panel). (B) Time‐course experiment of FBS re‐supplementation. PC3 (left panel) and MDA‐MB‐231 (middle panel) cells were serum starvation for 48 h prior to FBS re‐supplementation. Scale bar: 10 µm. Fifteen fields were acquired for each condition, and the mean fluorescence ratio of PMY/Ribosome P staining for each field was quantitated using ImageJ (right panel). (C) Newly synthesized proteins from isolated nuclei of pretreated PC3 cells as indicated were labeled with BioT and detected by western blotting using streptavidin‐HRP (left panel). RPL26 was used as a loading control. Quantification of BioT intensity was shown (mean ± SEM of triplicate experiments) (right panel). Data are shown as mean ± SEM. *p < 0.05, ***p < 0.005. Two‐tailed unpaired *t*‐test. PMY: puromycin; Ribo‐P: ribosomal P.

The tremendous difference in NT intensity between normal immortalized and cancer cells suggests that NT may relate to cancer cell growth. To test this idea, we determined the effects of growth suppression on NT in cancer cells. Serum starvation is a classic cellular stress that inhibits cell growth. Indeed, the growth of cancer cells, such as PC3 and MDA‐MB‐231, was positively correlated with the concentration of fetal bovine serum (FBS) (Figure S5). Accordingly, reduced NT was accompanied by decreasing the concentration of FBS, indicating a positive association between serum concentration, NT, and cancer cell growth (Figure 3A). To further verify this idea, we expanded FBS starvation in various cancer cells and found that it dramatically diminished nuclear PMY staining in additional 11 malignant cancer cell lines across multiple cancer types (Figure S3), suggesting that the consequence of serum starvation in NT is broadly conserved. Moreover, when cells were re‐supplemented with fresh FBS for 6 h, the PMY signals reappeared and intensified (Figure S3). Time‐course experiments on PC3 and MDA‐MB‐231 cells showed that the PMY signals were nearly recovered when cells were examined 2 h after FBS re‐supplementation (Figure 3B). This rapid recovery of NT suggests that NT may not merely be a byproduct of cell proliferation, but may contribute to cell growth.

In addition to NRPM, we also used a modified PUNCH‐P approach to demonstrate the association between FBS starvation or re‐supplementation and NT. Newly synthesized proteins from isolated nuclei of PC3 cells, pretreated as indicated, were labeled with BioT and detected by western blotting with streptavidin‐HRP. The analysis showed that FBS starvation reduced BioT signal intensity, while FBS re‐supplementation for 6 h greatly recovered BioT signal intensity, confirming the effect of serum status on NT (Figure 3C).

**FIGURE 4:**
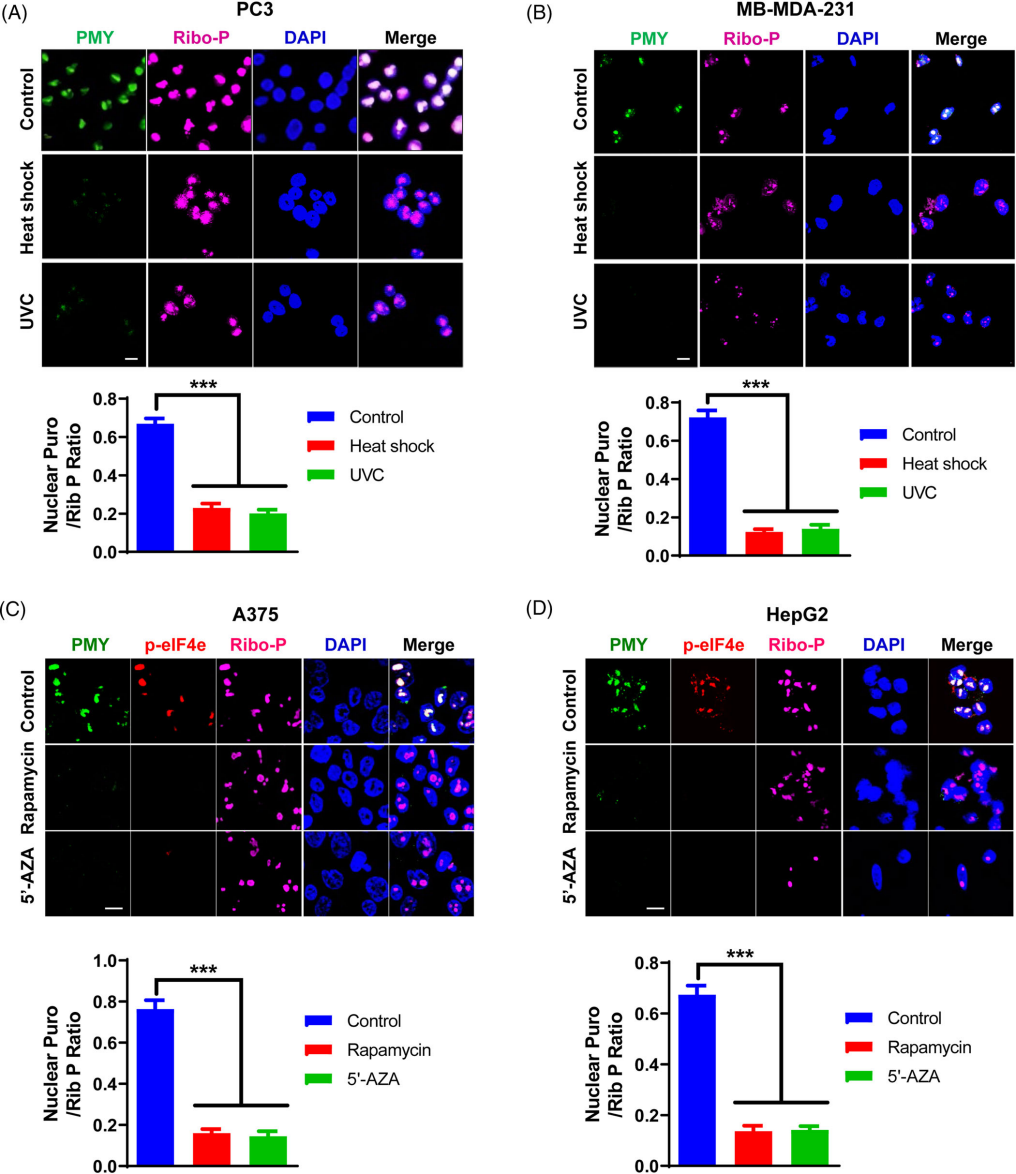
Reduced nuclear translation in malignant cancer cells by growth suppression and anti‐tumor agents. (A, B) PC3 (A) and MDA‐MB‐231 (B) cells were subjected to cellular stresses as indicated, followed by the nuclear ribopuromycylation (NRPM) analysis (upper panel). Scale bar: 10 µm. Fifteen fields were acquired for each condition, and the mean fluorescence ratio of PMY/Ribosome P staining for each field was quantitated using ImageJ (lower panel). UVC: ultraviolet‐C. (C, D) A375 (C) and HepG2 (D) cells were pretreated with rapamycin and azacitidine (5′‐AZA) respectively, followed by the NRPM analysis (upper panel). Scale bar: 10 µm. Fifteen fields were acquired for each condition, and the mean fluorescence ratio of PMY/Ribosome P staining for each field was quantitated using ImageJ (lower panel). Data are shown as mean ± SEM. ***P < 0.005. Two‐tailed unpaired *t*‐test. PMY: puromycin; Ribo‐P: ribosomal P.

Similarly, ultraviolet‐C (UVC) radiation and heat shock, two other known cellular stresses repressing cell growth, markedly diminished NT in PC3 and MDA‐MB‐231 cells (Figure 4A,B). Furthermore, we explored the potential relationship between anti‐tumor agents and NT. Rapamycin, a specific inhibitor of mTOR that is a central regulator of metabolism and cell growth, has been extensively demonstrated to be useful in the treatment of various diseases, including cancer.^32^ Azacitidine (5′‐AZA), an inhibitor of DNA methyltransferases, has been approved by the Food and Drug Administration as an anti‐tumor drug.^33^ Interestingly, NRPM analyses showed both rapamycin and 5′‐AZA treatments markedly abolished NT in cancer cells, such as A375 and HepG2 (Figure 4C,D). Taken together, these results demonstrate that both cell stresses and anti‐tumor agents can reduce NT, indicating potential involvement of NT in cancer cell growth.

### 2.4 PUNCH‐P analysis of actively translated proteins in PC3 nuclei

The ribosome dependence of NT provides an opportunity to use ribosome profiling to identify the nascent peptides translated into the nucleus.^34^ Therefore, we modified the PUNCH‐P approach that combines biotinylated puromycin with MS analysis to globally monitor mRNA translation. The modified PUNCH‐P involves the isolation of nuclei followed by regular procedures, including ribosome isolation, biotinylated PMY labeling, streptavidin affinity purification, and liquid chromatography‐tandem mass spectrometry (LC‐MS/MS) (Figure S6).

**FIGURE 5:**
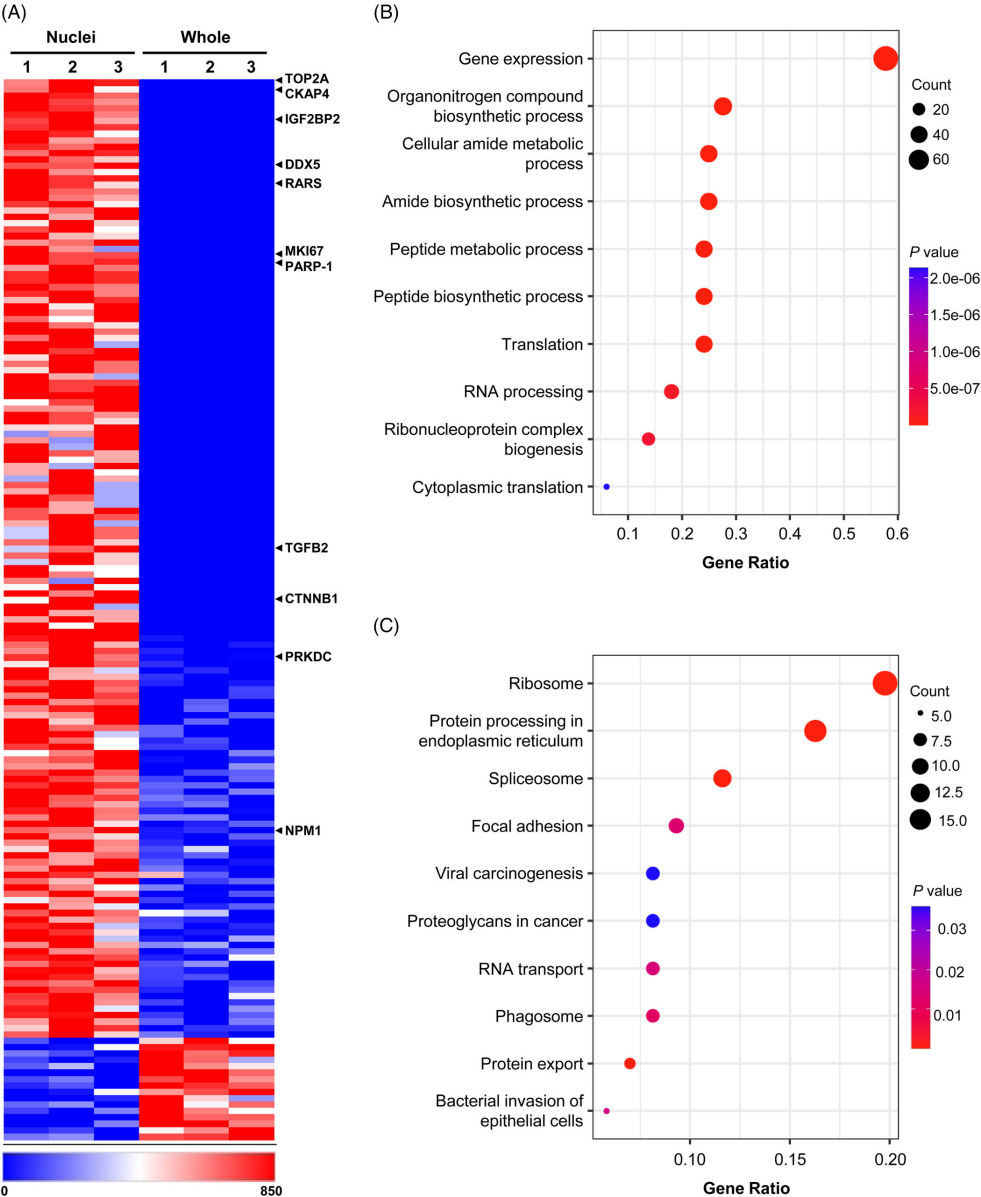
Puromycin‐associated nascent chain proteomics (PUNCH‐P) analysis of PC3 nuclei. (A‐C) Whole‐cell extracts or isolated nuclei of PC3 cells were labeled by BioT. Equal amounts of samples were subjected to LC‐MS/MS analysis. (A) Heat map of newly synthesized proteins with statistically significant differences in PC3 whole‐cell extracts or isolated nuclei (Fold > 1.5, *p* < 0.05). Red and blue depict higher and lower protein levels, respectively, and color intensity indicates the magnitude of expression differences. Selected NT‐derived oncoproteins are indicated on the right. (B) Gene ontology (GO) analysis on proteins that are enriched in nuclei. (C) KEGG analysis on proteins enriched in nuclei.

Modified PUNCH‐P analysis on newly‐synthesized proteins within the whole‐cell extracts or isolated nuclei of PC3 cells found that a subclass of 150 proteins was preferentially translated in nuclei (Fold > 1.5, *p* < 0.05) (Figure 5A and Table S2). Among them, 134 proteins (89.3%) have been shown to have oncogenic activity (Figure 5A and Table S2), including a number of well‐known oncogenes such as TOP2A (topoisomerase IIα), PARP‐1 (poly (ADP‐ribose) polymerase‐1), MKI67 (marker of proliferation Ki‐67), CTNNB1 (catenin beta 1), and PRKDC (protein kinase, DNA‐activated, catalytic polypeptide). TOP2A, an essential regulator for separating replicated chromosomes and a proven therapeutic target of various cancers,^35^ showed the highest enrichment in the NT. These results suggest an enrichment of oncoproteins resulting from NT.

We then analyzed these NT candidates using the DAVID gene ontology (GO) program^36^ and found that they were significantly involved in fundamental cellular processes, such as translation, peptide metabolism, gene expression, and RNA processing (Figure 5B and Table S3). Notably, 67 proteins (45% of total NT‐derived candidates) were predicted to have a role in gene expression, indicating NT might substantially modulate the transcriptome of cancer cells. KEGG analysis further showed that NT‐derived candidates are involved in several processes related to tumorigenesis, including focal adhesion, proteoglycans in cancer, and viral carcinogenesis, further confirming the potential role of NT in cancer development (Figure 5C and Table S4). Taken together, these results confirm the presence of active NT in cancer cells, and indicate that NT‐derived proteins are involved in tumorigenesis.

### 2.5 Nuclear expression of oncogenic mRNA increases protein translation and promotes tumorigenesis

**FIGURE 6:**
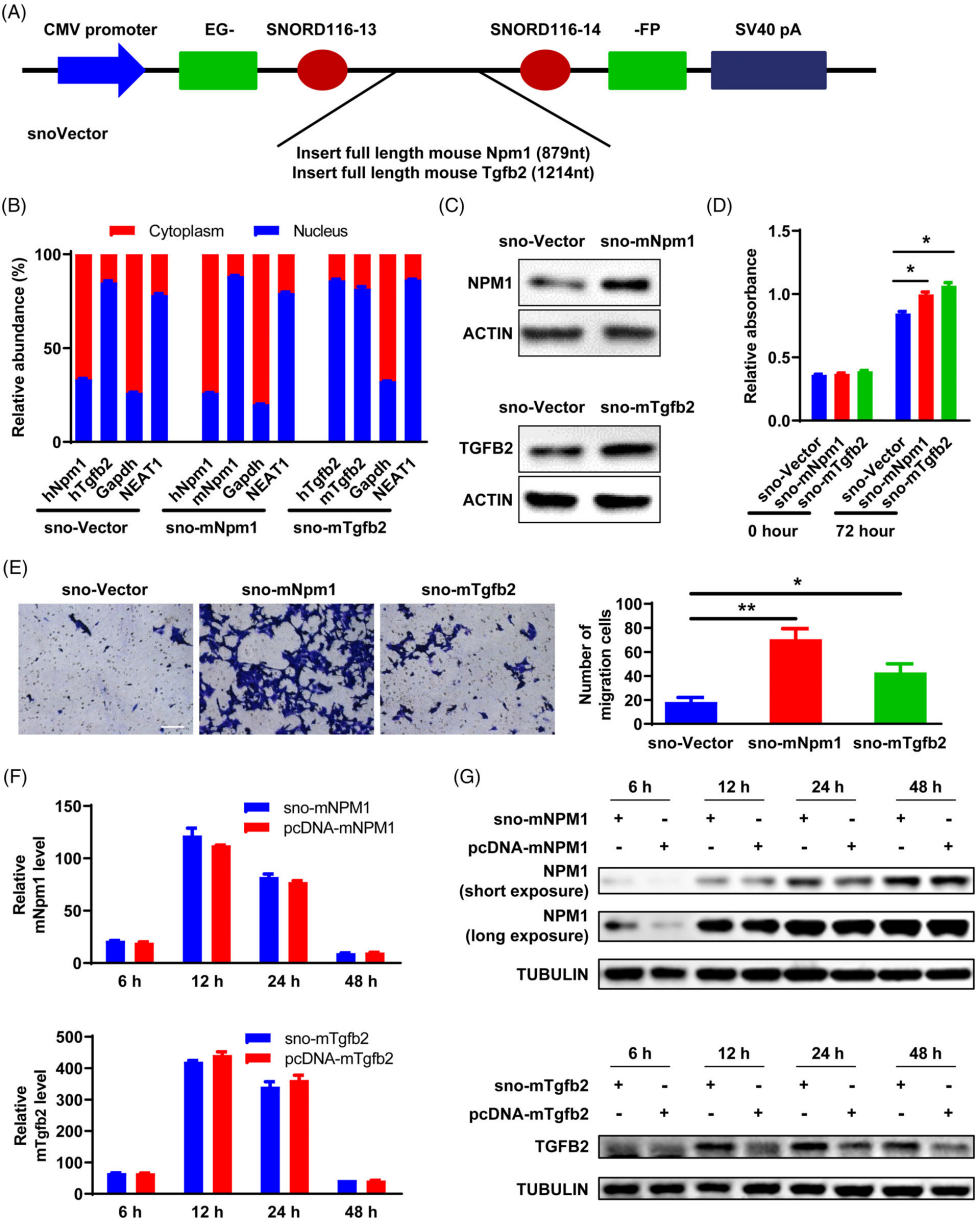
SnoVectors expressing oncogenic mRNA in the nucleus increase its protein levels and promote cancer cell growth and migration. (A) A schematic view of the snoVector that can lead to the nuclear retention of inserted RNA sequences. (B–E) HCT116 cells were transfected with indicated snoVectors, and the following analyses were performed. (B) Subcellular distribution of transfected RNAs from different snoVectors at 24 h post‐transfection. (C) Protein expression in whole‐cell extracts was measured by western blotting at 48 h post‐transfection. (D) Cell proliferation was determined by the MTS analysis at 72 h post‐transfection. (E) Cell migration was assessed by transwell assays. Scale bar: 100 µm. Quantification of migration cells was shown (right panel). (F and G) HCT116 cells were transfected with indicated snoVectors and pcDNA vectors, respectively. mRNA (F) and protein (G) levels of indicated genes in whole‐cell extracts were determined at different time points post‐transfection. Data are shown as mean ± SEM (triplicate experiments). *p < 0.05, **p < 0.01. Two‐tailed unpaired *t*‐test.

Recently, Yin et al. developed snoVector, an expression vector that can express interested RNAs and constrain their accumulation in the nucleus.^37^ We adopted this vector system to verify the existence of NT and its potential functional consequences. To this end, we introduced into the snoVector the full‐length sequence of mouse Nmp1 (mNmp1) and Tgfb2 (mTgfb2) (Figure 6A), two oncogenes that are preferentially translated in nuclei as identified by our nuclear PUNCH‐P analysis (Figure 5A). This design allowed us to discriminate the snoVector‐expressed mouse RNAs from the endogenous human homologs after introduction into human cells. In HCT116 cells, human Nmp1 (hNmp1) transcripts were mainly detected in the cytoplasm, while nearly 90% of snoVector‐expressed mNmp1 transcripts were accumulated in the nucleus, as revealed by RNA fractionation followed by quantitative reverse‐transcriptase polymerase chain reaction (qRT‐PCR) (Figure 6B). Unlike hNpm1, hTgfb2 transcripts were mainly accumulated in the nucleus and snoVector‐expressed mTgfb2 transcripts exhibited similar nuclear localization. These results demonstrated that exogenous mRNA transcripts by snoVector are predominantly restricted in the nucleus, providing an excellent approach for testing NT. Intriguingly, nuclear accumulation of mNpm1 and mTgfb2 transcripts led to an increase in their corresponding protein levels in HCT116 and PC3 cells (Figure 6C and Figure S7), which may partially result from NT.

Both NPM1 and TGFB2 have been shown to promote cancer cell growth and metastasis,^38^, ^39^ we thus determined whether snoVector‐expressed oncoproteins exert these oncogenic activities. MTS assays revealed that snoVector‐expressed Nmp1 and Tgfb2 enhanced cell proliferation (Figure 6D). Moreover, transwell analyses showed that the number of migrated cells was higher in cells transfected with snoVector‐mNmp1 or ‐mTgfb2 than those in control cells (Figure 6E), suggesting an increase in cell migration.

In a conventional view, the production of protein from mRNAs is a complex and time‐consuming process that is tightly regulated at many levels, including mRNA transport from the nucleus to the cytoplasm. In this regard, we wondered whether NT has a higher rate of protein production than conventional cytoplastic translation. To test this idea, we additionally established pcDNA constructs containing the full‐length sequence of mNmp1 and mTgfb2 respectively, which can achieve regular CT and were used to compare with the above snoVectors. We optimized the parameters of transfection and achieved similar mRNA levels of inserted genes in HCT116 cells transfected snoVector and pcDNA vector, respectively (Figure 6F). In this circumstance, snoVector‐transfected cells exhibited an obvious increase in NPM1 and TGFB2 protein levels compared with pcDNA‐transfected cells, especially at early time points post‐transfection (Figure 6G), indicating that nuclear‐retained mRNAs might offer potential for fast protein production. Taken together, these results suggest that nuclear expression of oncogenes could improve protein translation and promote tumorigenesis.

## 3 DISCUSSION

The present study demonstrates unexpectedly high levels of NT in cancer cells. NT is thought to be a rare event because most normal cells have active CT. Here we find that NT is scarce in normal immortalized cells, but is ubiquitous and robust in malignant cancer cells. Moreover, NT is under dynamic regulation by growth signals, suggesting a positive association between NT and cell proliferation/growth.

In this study, we performed NRPM to determine NT in 18 malignant cancer cell lines across seven cancer types, as well as in 4 normal immortalized cell lines. Moreover, a combination of other approaches, including PUNCH‐P and snoVector, were used to verify the enhancement of NT in malignant cells, and identify potential NT‐derived proteins and their oncogenic functions. These data collectively suggest that NT is dramatically increased in cancer cells and associated with cancer cell growth and progression. However, both NRPM and PUNCH‐P could not exclude the possibility of cytoplasmic contamination.^18^ Similarly, a small portion of exogenous mRNA transcripts mediated by snoVector were also presented in the cytoplasm. In this regard, it is important for future studies to further validate the presence and functional outcome by using improved NRPM and PUNCH‐P, and/or newly developed techniques tracking translation in vivo. In addition, future investigations are required to further confirm the significance of NT in tumorigenesis. For instance, the existence and function of NT should be verified in primary cancer cells, together with paired normal cells, from human patients. It is also of interest to determine the stability and homogeneity of NT in the cancer cell population, as well as the oncogenic outcome of NT‐derived protein in animal models.

Although it is currently unknown whether enhanced NT is a cause or consequence of cancer cell growth, our findings provide some clues for this enduring enigma. On the one hand, cancer cells contain large chromosomal rearrangements that might induce various surveillance pathways (such as NMD) in the nucleus,^40^ thereby increasing NT. This notion is consistent with the important role of NMD in cancer development,^24^, ^27^ as well as the observation that the majority of nucleolus‐associated proteins identified by proteomic analyses are encoded by as‐yet uncharacterized open reading frames.^41^ On the other hand, it is also likely that NT could speedily generate proteins to spur the rapid growth of cancer cells. In this regard, NT is similar to compartmental translation (also known as local translation), which has emerged as a new regulatory mechanism to facilitate specific biological functions.^42^ Of note, translation can be coupled to transcription in prokaryotes. In eukaryotes, transcription and translation are physically separated and are conventionally uncoupled to ensure the fidelity of genetic information, although this intricate and precise regulation is time‐consuming. It is reasonable to speculate that cancer cells may adopt NT to maintain their rapid growth at the expense of genetic fidelity. In line with this possibility, many cancerous proteins that are preferentially translated in the nucleus were identified by the modified PUNCH‐P approach. Recent studies also provide some clues to support this possibility. Apcher et al. showed that NT of pre‐spliced RNAs generates antigenic peptides for the MHC class I pathway.^4^ Additionally, it has been proposed that repeat‐associated non‐ATG (RAN) translation of C9orf72 in the nucleus may contribute to human diseases, such as amyotrophic lateral sclerosis and frontotemporal dementia.^43^ Furthermore, nuclear accumulation of oncogenes in cancer cells by snoVector can lead to increased protein levels and enhanced tumorigenic activities, indicating a potential function of NT‐derived oncoproteins.

We hope our studies will catalyze increased interest in NT. For example, the robust and ubiquitous NT in cancer cells provides an excellent model to study NT itself. A better understanding of NT will greatly extend our understanding of protein translation and the multifunctional nucleolus. In particular, it is of great interest for future investigation to explore whether NT enhances and complements cytoplasmic protein translation or produces specific proteins for special cancerous demands, which will offer invaluable information on molecular mechanisms underlying tumorigenesis. It is highly anticipated that recent technological advances, such as imaging approaches tracking translation in vivo,^44^ will benefit and promote the dissection of NT in the near future. Finally, given that NT is much higher in cancer cells than normal immortalized cells, NT may represent an ideal target for cancer treatment.

In summary, this work demonstrates that NT is robustly increased in malignant cells and positively correlated with cancer cell growth and progression. These findings suggest a previously unknown pathological mechanism of tumorigenesis and would eventually offer attractive therapeutic strategies for the treatment of cancer.

## 4 MATERIALS AND METHODS

### 4.1 Cell culture and treatments

All human cancer cell lines including 786‐O (kidney carcinoma), A375 (melanoma), A549 (lung carcinoma), Caco‐2 (colorectal adenocarcinoma), Caki‐1 (kidney carcinoma), Caki‐2 (kidney carcinoma), HCT8 (colorectal adenocarcinoma), HCT116 (colorectal adenocarcinoma), HT‐29 (colorectal adenocarcinoma), Hep3B (liver carcinoma), HepG2 (liver carcinoma), MDA‐MB‐231 (breast adenocarcinoma), MHCC97H (liver carcinoma), MHCC97L (liver carcinoma), SK‐HEP‐1 (liver carcinoma), PC‐3 (prostate adenocarcinoma), SK‐MEL‐5 (melanoma), SNU395 (liver carcinoma), SNU398 (liver carcinoma), and normal immortalized cell lines including CL‐48 (hepatocytes), HUVEC (endothelial cells), IMR‐90 (lung fibroblast) and MRC‐5 (lung fibroblast) were obtained from ATCC (American Type Culture Collection) and JCRB (Japanese Collection of Research Bioresources). These cell lines were grown in RPMI‐1640 supplemented with 10% FBS, 100 Units/ml penicillin, and 100 µg/ml streptomycin in a humidified CO_2_ incubator at 37˚C. For the induction of cellular stresses that prevented cell proliferation, cells were incubated in a serum‐depleted RPMI‐1640 medium for 48 h. For the UVC stress, cells were irradiated with 10−100 J/m^2^ UVC and then allowed to recover in a complete medium for 6 h. For heat shock, cells were incubated at 44˚C for 2 h.

### 4.2 RPM

Regular and nuclear RPM staining was conducted as previously described with minor modifications.^2^ Cancer or normal immortalized cells were seeded on coverslips in 6 well plates the day before nuclear RPM staining. Cells were PMY‐labeled using RPMI‐1640 medium supplemented with 10% FBS, penicillin/streptomycin, 91 µM PMY (Sigma), and 208 µM emetine (EMD Millipore) for 5 min at 37˚C. The cytoplasmic fraction was extracted using prechilled permeabilization buffer containing 50 mM Tris‐HCl, pH 7.5, 150 mM NaCl, 1% IGEPAL CA‐630 (v/v) (Sigma), and EDTA‐free protease inhibitor (Roche) for 5 min on ice. Cells were briefly washed with permeabilization buffer without IGEPAL CA‐630 and were fixed with 3% paraformaldehyde (Sigma) for 15 min at room temperature. For immunofluorescence staining of puromycylated peptides tethered in nuclear ribosomes, fixed cells were incubated in blocking buffer (1% bovine serum albumin in phosphate‐buffered saline [PBS]) containing mouse anti‐PMY monoclonal antibody (12D10, EMD Millipore), rabbit monoclonal anti‐fibrillarin antibody (2639S, Cell Signaling Technology), rabbit polyclonal anti‐nucleolin antibody (ab22758, Abcam), rabbit monoclonal anti‐phospho‐eIF4e (p‐eIF4e) antibody (ab76256, Abcam) and human anti‐ribo P antibody (HPO‐0100, ImmunoVision) overnight at 4˚C. After washing three times in PBS, cells were incubated in a blocking buffer containing Alexa Fluor 488 Goat Anti‐Mouse IgG (H+L) Antibody (A‐11001, Life Technologies), Alexa Fluor® 594 Goat Anti‐Rabbit IgG (H+L) Antibody (A11037, Life Technologies), and Cy5‐AffiniPure Donkey Anti‐Human IgG (H+L) (AB‐2340539, Jackson ImmunoReserach) for 1 h at room temperature. The cells were then washed in PBS three times and nuclei were counter‐stained with the nuclear counterstain 4′,6‐diamidino‐2‐phenylindole (DAPI, 0.2 µg/ml) for 10 min at room temperature. The cells were mounted on slides using Vectashield (Vector). All images were acquired using a Zeiss LSM510 META NLO Axiovert 200 M Inverted Confocal Microscope and analyzed using the LSM browser software. Each set of images for a given experiment was processed identically to maintain the image intensity ratio. ImageJ and Prism software was used for quantitation.

### 4.3 PUNCH‐P

Regular PUNCH‐P analyses of total polyribosomes in whole cells were conducted as previously described.^34^ To identify puromycin‐associated nascent peptide chains in the nuclear polyribosome, nuclear PUNCH‐P analyses were performed as follows. Nuclei were fractionated from PC‐3 cells using the Nuclei EZ Prep Kit (Sigma) according to the manufacturer's instructions. Isolated nuclei and intact PC‐3 cells were lysed for 20 min in a polysome buffer containing 20 mM Tris‐HCl, pH 7.5, 20 mM KCl, 10 mM MgCl_2_, 10 mM NaF, 10 mM α‐glycerolphosphate, 2 µg/ml pepstatin A (Sigma), 2 µg/ml leupeptin (Sigma), Complete EDTA‐free protease inhibitor cocktails (Roche), 1.25 mM dithiothreitol, 200 U/ml SUPERase·In RNase inhibitor (Invitrogen), 1% Triton X‐100 and 1% deoxycholate. Nuclear and total lysates were centrifuged at 14,000 rpm for 10 min at 4˚C and the supernatant was layered on 1 M sucrose cushions and centrifuged at 35,000 rpm at 4˚C for 4 h in a Thermo Scientific Swinging Bucket Rotor (Thermo Scientific). Pellets were resuspended and puromycylated in polysome buffer containing Biotin‐dC‐PMY (Jena Bioscience) for 10 min at 37˚C. The incorporation of puromycin on polyribosomes was stopped with a high‐stringency washing buffer containing 100 mM Tris‐HCl, pH 7.5, 2% SDS, 8 M urea, and 150 mM NaCl. Puromycin‐associated nascent peptide chains were captured on High Capacity Streptavidin Agarose beads (Pierce) with rotation overnight at room temperature and then were briefly washed in high stringency washing buffer four times, followed by washing in the same buffer for 30 min at room temperature. The beads were further washed for 30 min in a high salt buffer containing 100 mM Tris‐HCl, pH 7.5, and 150 mM NaCl, then washed in ultra‐pure water five times. The puromycylated peptides were denatured in 1 mM dithiothreitol and 50 mM iodoacetamide, followed by washing in 50 mM ammonium bicarbonate twice. For LC‐MS/MS analysis, the puromycylated peptides were digested by sequencing grade‐modified trypsin (Promega) overnight and acidified with 0.1% TFA. The digested peptides were concentrated and desalted by C18 Tips (Pierce). LC‐MS/MS analysis for PUNCH‐P samples was performed by Stanford University Mass Spectrometry Core Facility. This analysis identified that 150 proteins were preferentially translated in PC3 nuclei compare with PC3 whole cells. The association of these proteins with tumorigenesis was assessed by searching literature indexed in the PubMed database. For Western blot analysis, the puromycylated peptides released from beads were boiled and denatured in elution buffer containing 2% SDS, 3 mM biotin, and 8 M urea in PBS.

### 4.4 Western blot

The puromycylated peptides samples were electrophoresed on 10% SDS polyacrylamide gels and were transferred into PVDF membranes (Bio‐Rad). The membranes were stained using ponceau S (Sigma), washed in pure water for 10 min, and photographed. The membrane was blocked in a 5% milk solution and immunoblotted in a blocking solution containing Pierce High Sensitivity Streptavidin HRP Conjugate (Pierce). The membrane was washed in TBST three times for 5 min each and was developed in Pierce ECL Western Blotting Substrate (Pierce). Regular western blot analysis was performed as described previously.^45^ Antibodies for western blot analysis were purchased from Cell Signaling Technology (anti‐Fibrillarin, 2639S; anti‐GAPDH, 5174S; anti‐HSP90, 4877S; anti‐Jun, 9165S; anti‐NPM1, 3542S; anti‐PDI, 2446S; anti‐RPL26, 2065S), Abclonal Technology (anti‐TGFB2, A3640), and Proteintech (anti‐Tubulin, 10068‐1‐AP), respectively.

### 4.5 Plasmid construction

pZW1‐snoVector, which can stably express the gene of interest and constrain its RNA molecules within the nucleus,^37^ was kindly provided by Dr. Lingling Chen (Chinese Academy of Sciences, Shanghai, China). Coding regions for Npm1 and Tgfb2 were amplified from mouse cDNA and then cloned into pZW1‐snoVector and pcDNA3.1 vector, respectively. Two small nucleolar RNA (snoRNA) genes SNORD116‐13 and SNORD116‐14 and an interested mRNA coding region (Nmp1 or Tgfb2) were inserted into the intron of Enhanced Green Fluorescence Protein. The correct orientation and integrity of the construct were confirmed by sequencing. All primers for plasmid construction were listed in Table S1.

### 4.6 Transfection

Cells were cultured in a 6‐well plate and transfected with plasmids using Attractene (QIAGEN) as described previously.^46^ 48 h post‐transfection, cells were collected for RNA or protein isolation.

### 4.7 Cell proliferation assay

Cell proliferation was measured by MTS (3‐[4,5‐dimethylthiazol‐2‐yl]−5‐[3‐carboxymethoxyphenyl]−2‐[4‐sulfophenyl]−2*H*‐tetrazolium) assay as described previously.^45^ In brief, cells were seeded in a 96‐well plate, and rates of cell proliferation were measured by using the CellTiter 96 AQ_ueous_ Nonradioactive Cell Proliferation Assay (Promega).

### 4.8 Cell migration assay

Transwell migration assay was performed as previously described.^45^ Briefly, 50,000 cells were seeded in the upper chambers of the transwell plates with FBS‐free media and incubated for 24 h. Then cells on the lower surface of the membrane were fixed, stained, and photographed by an optical microscope.

### 4.9 Analysis of nuclear and cytoplasmic RNA abundance

Nuclear and cytoplasmic RNA was isolated as previously described^47^ with minor modification. Cells were harvested, centrifuged at 1000 rpm for 8 min, and washed twice with ice‐cold PBS. Cell pellets were resuspended in 200 µl lysis buffer A (5 mM Tris (pH 8.0), 140 mM NaCl, 1.5 mM MgCl_2_, 0.5% CA‐630, 40 U Ribonuclease Inhibitor), incubated on ice for 5 min, and centrifuged at 1000 g for 3 min at 4°C to obtain the cytoplasmic supernatants and the nuclear pellets. Nuclear pellets were further washed twice with 50 µl lysis buffer A before RNA extraction. Equal amounts of cytoplasmic and nuclear samples were used for RNA isolation by Trizol (Invitrogen). The cDNA was transcribed with MMLV (Invitrogen) with random hexamers. Gene expressions were measured by qRT‐PCR as described previously.^48^ Primers for qRT‐PCR were listed in Table S1.

### 4.10 Statistical analysis

Numerical values are reported as mean ± SEM. Statistical significance (*p ≤ 0.05, **p ≤ 0.01, and ***p ≤ 0.005) was determined by a two‐tailed unpaired *t*‐test.

## AUTHOR CONTRIBUTIONS

Xianghui Fu, Wendong Huang, Byung‐Wook Kim, and Sailan Zou. designed research; Sailan Zou, Byung‐Wook Kim. Xianghui Fu, Jiawei Zhang, Geng Liu, and Yan Tian. performed research; Xianghui Fu, Wendong Huang, Byung‐Wook Kim, Sailan Zou, Guixiang Zhang, Xiao Du, Ricardo Zerda, Zhuo Li, Weiqiang Lin, and Xiang Gao. analyzed data; and Byung‐Wook Kim, Wendong Huang, Xianghui Fu, and Yan Tian. wrote the paper. All authors have read and approved the final manuscript.

## CONFLICT OF INTEREST STATEMENT

The authors declare no conflict of interest.

## ETHICS STATEMENT

Not applicable.

## Supporting information

Supporting Information

## Data Availability

The data that support the findings of this study are available from the corresponding author upon reasonable request.
