# Supplementary material for: Enhanced nuclear translation is associated with proliferation and progression across multiple cancers
Source: MedComm (2020). 2023 Apr 14;4(3):e248. doi: 10.1002/mco2.248 (PMC10104727; doi:10.1002/mco2.248)
Supplement: Supplementary file 1 — Supporting Information [file MCO2-4-e248-s001.pdf]

Enhanced nuclear translation is associated with proliferation and progression across multiple cancers

Sailan Zou[1,#], Byung-Wook Kim[2,#], Yan Tian[1,#], Geng Liu[1], Jiawei Zhang[2,3], Ricardo Zerda[4], Zhuo Li[4], Guixiang Zhang[5], Xiao Du[5,6], Weiqiang Lin[7], Xiang Gao[8], Wendong Huang[2,*], Xianghui Fu[1,*]

[1]Division of Endocrinology and Metabolism, State Key Laboratory of Biotherapy and Cancer Center, West China Hospital, Sichuan University and Collaborative Innovation Center of Biotherapy, Chengdu 610041, Sichuan, China.

[2]Department of Diabetes Complications and Metabolism, Arthur Riggs Diabetes and Metabolism Research Institute | Irell & Manella Graduate School of Biological Sciences, Beckman Research Institute, City of Hope National Medical Center, Duarte, CA 91010, USA.

[3]Cancer Institute (Key Laboratory of Cancer Prevention and Intervention, China National Ministry of Education), Second Affiliated Hospital, School of Medicine, Zhejiang University, 310009, Hangzhou, China.

[4]Electron Microscopy and Atomic Force Microscopy Core, City of Hope National Medical Center, Duarte, CA 91010, USA.

[5]Division of Gastrointestinal Surgery, Department of General Surgery and Gastric Cancer Center, West China Hospital, Sichuan University, Chengdu 610041, Sichuan, China.

[6]Department of General Surgery, Yaan People's Hospital, Yaan 625000, Sichuan, China.

[7]Department of Nephrology, The Fourth Affiliated Hospital, International Institutes of Medicine, School of Medicine, Zhejiang University, 310009, Zhejiang, China.

[8]Department of Neurosurgery and Institute of Neurosurgery, State Key

Laboratory of Biotherapy and Cancer Center, West China Hospital, West China Medical School, Sichuan University and Collaborative Innovation Center for Biotherapy, Chengdu 610041, Sichuan, China.

[#]These three authors contributed equally.

*Correspondence: whuang@coh.org (W.H.), xfu@scu.edu.cn (X.F.).

Email address: zsl96hn@163.com (S.Z.), BKim@coh.org (B.Kim.), tyfxh@163.com (Y.T.), liu2geng6@163.com (G.L.), jwzhang@zju.edu.cn (J.Z.), rzerda@coh.org (R.Z.), zhouli@coh.org (Z.L.), zhangguixiang@wchscu.cn (G.Z.), Duxiao_home@163.com (X.D.), wlin@zju.edu.cn (W.L.), xianggao@scu.edu.cn (X.G.).

Figure S1

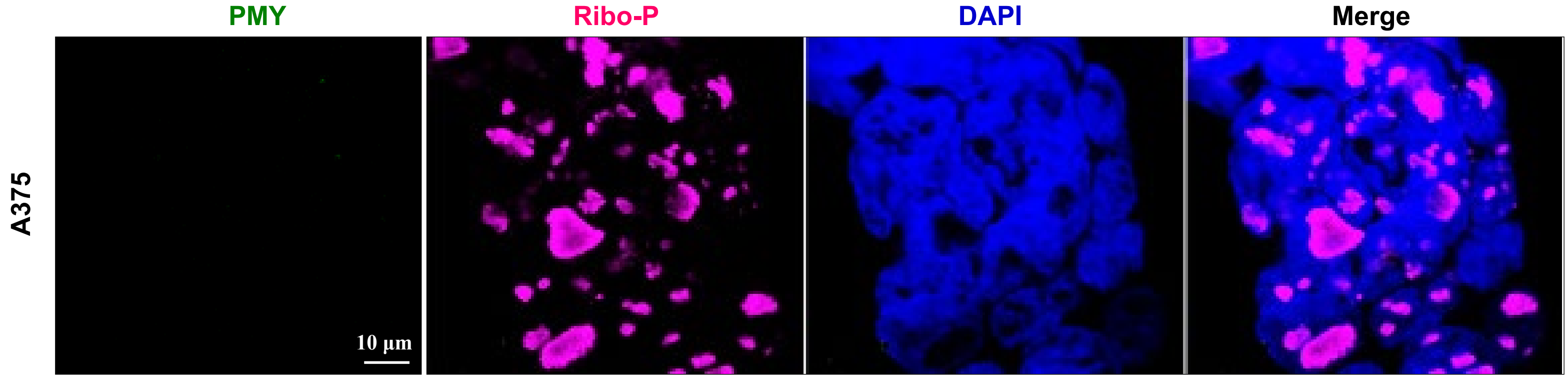

Figure S1. **Verification of RPM analysis.**

A375 cells without puromycin (PMY) treatment have no signal.

Figure S2

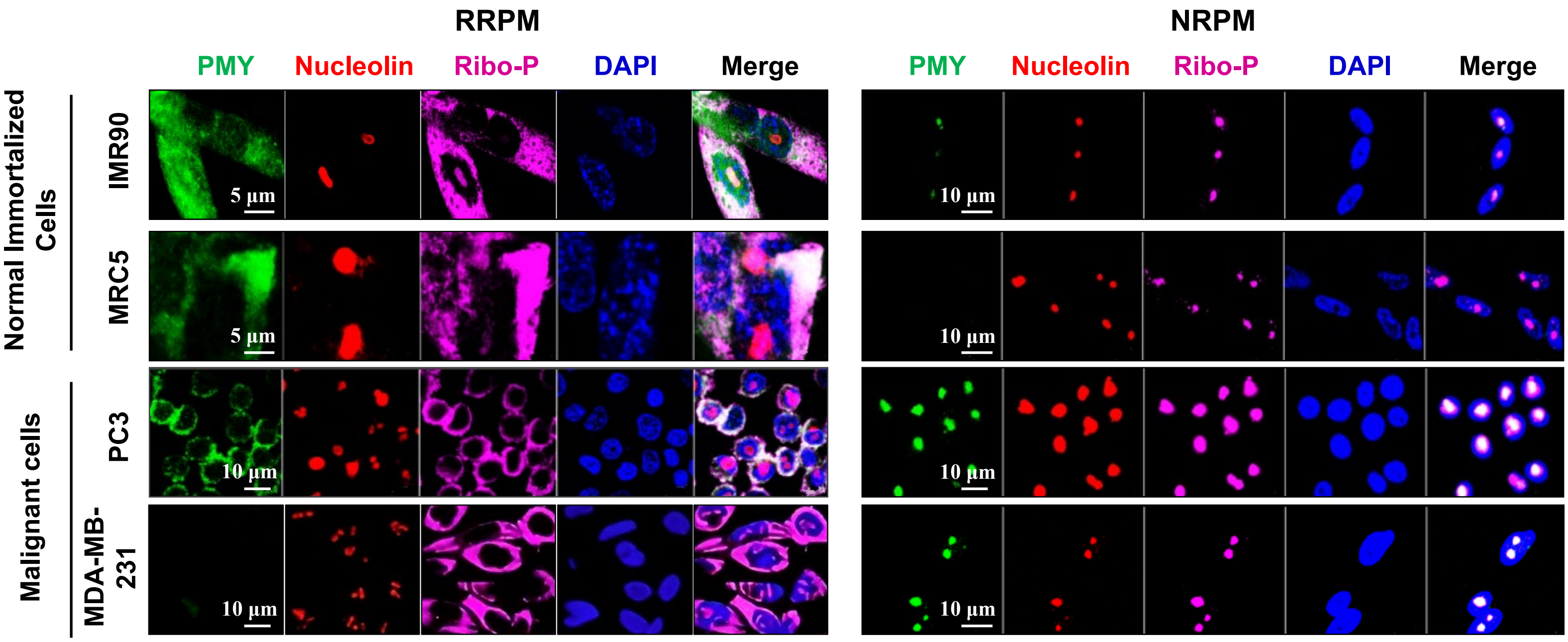

Figure S2. Regular RPM (RRPM) and nuclear RPM (NRPM) detect cytoplasmic and nuclear translation, respectively.

Normal immortalized cells (IMR90 and MRC5) and malignant cancer cells (PC3 and MDA-231) were used for regular RPM (RRPM) and nuclear RPM (NRPM), respectively. Nuclei are counterstained with DAPI.

Figure S3

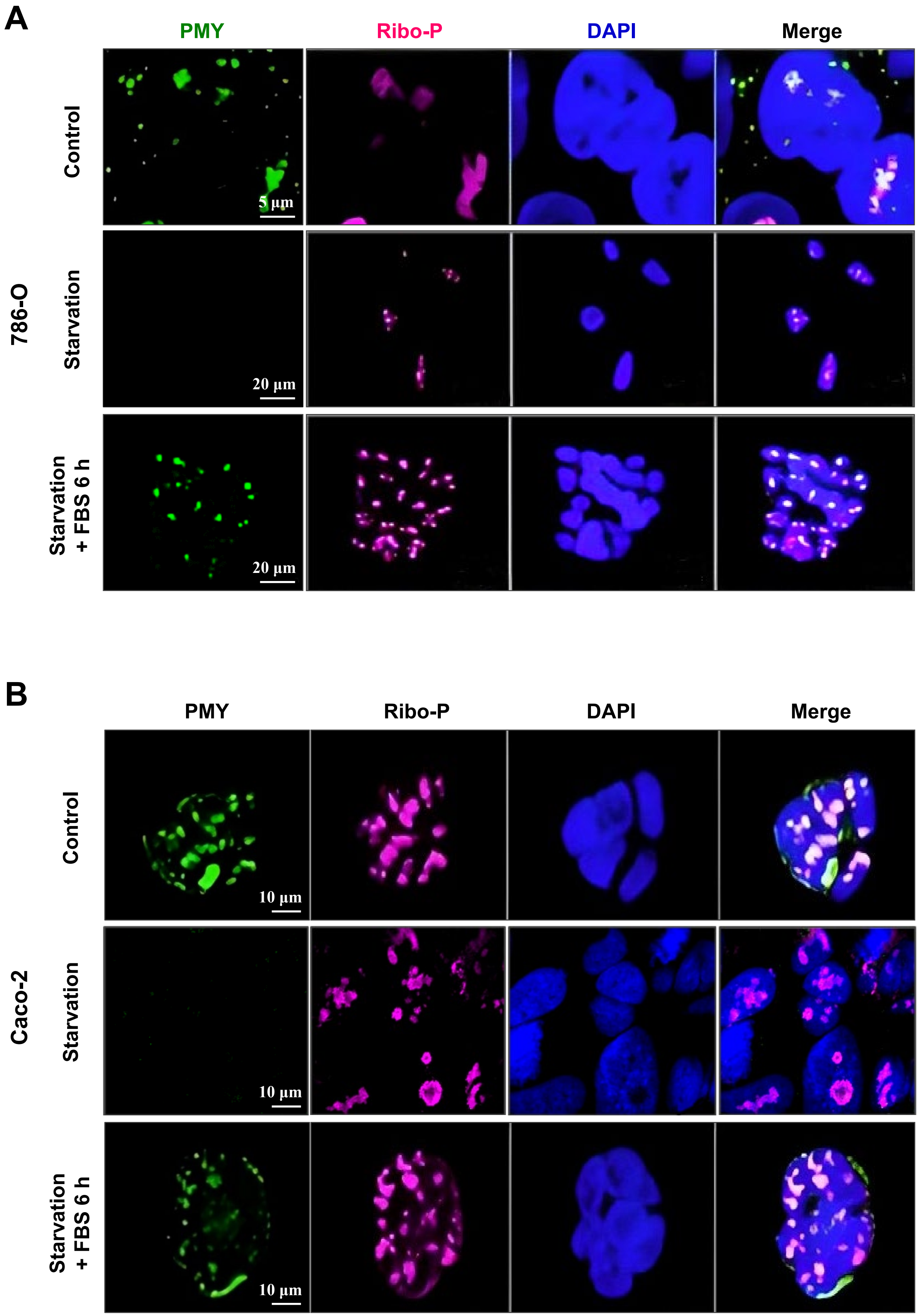

Figure S3. **Nuclear translation detected by NRPM in various malignant cancer cell lines with or without serum starvation.**

Starvation + FBS 6 h: cells were treated with serum starvation for 48 hours prior to FBS re-supplementation for 6 hours.

Figure S3

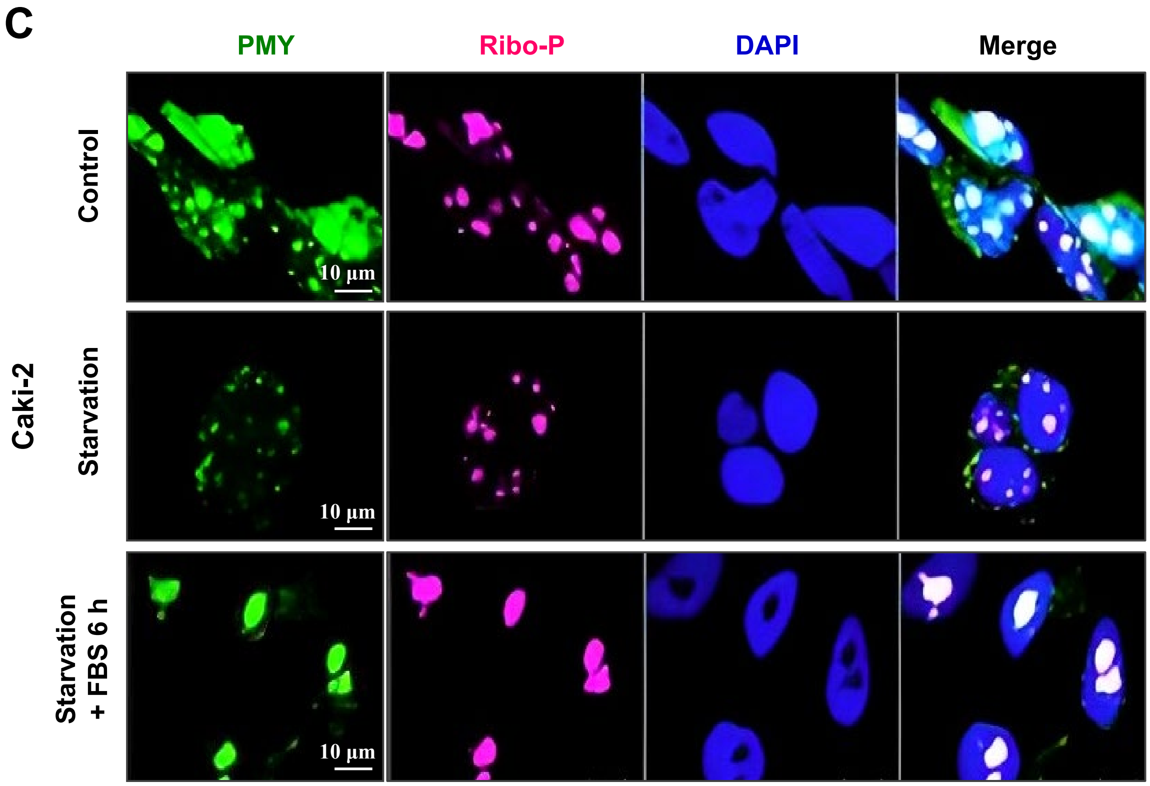

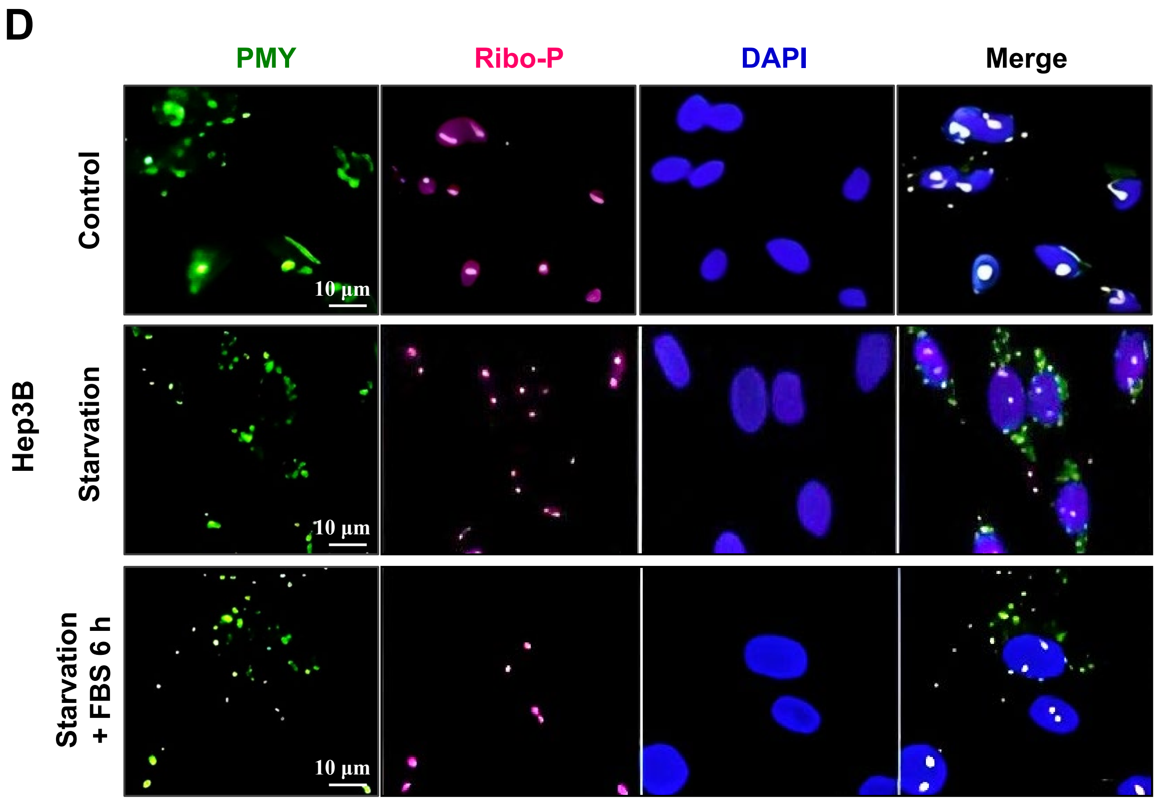

Figure S3. Nuclear translation detected by NRPM in various malignant cancer cell lines with or without serum starvation.

Starvation + FBS 6 h: cells were treated with serum starvation for 48 hours prior to FBS re-supplementation for 6 hours.

Figure S3

E

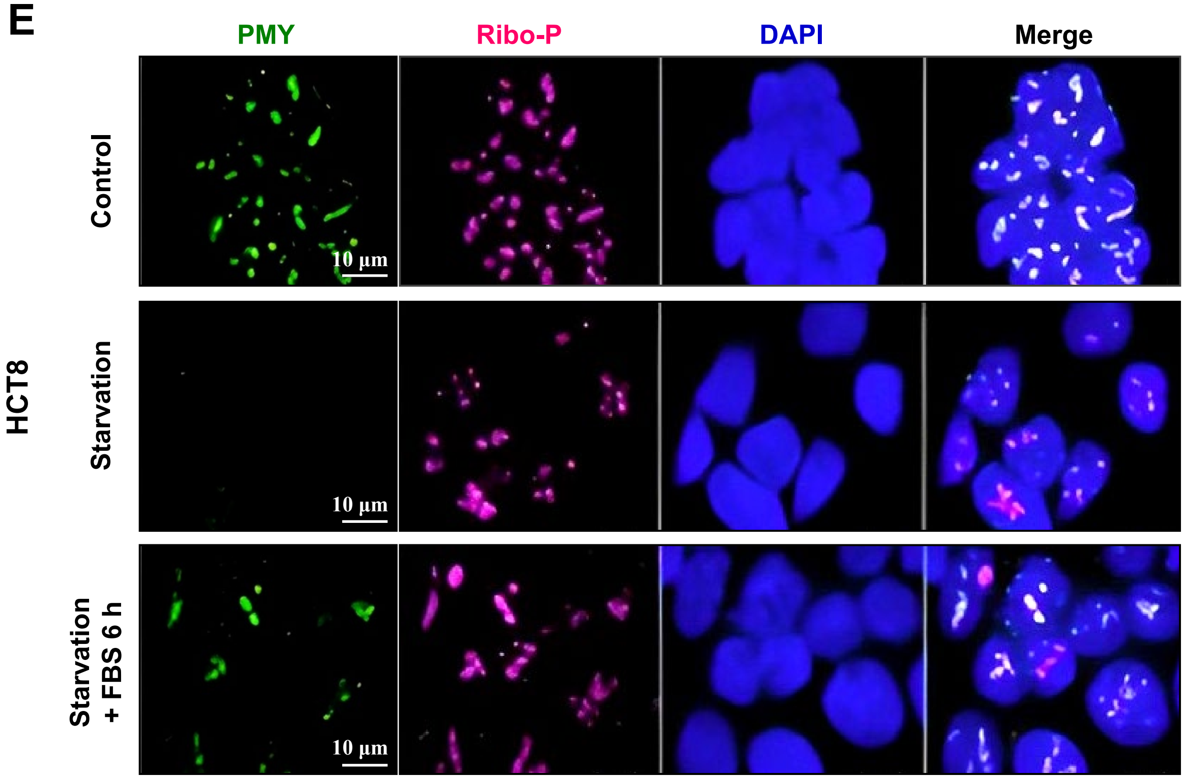

F

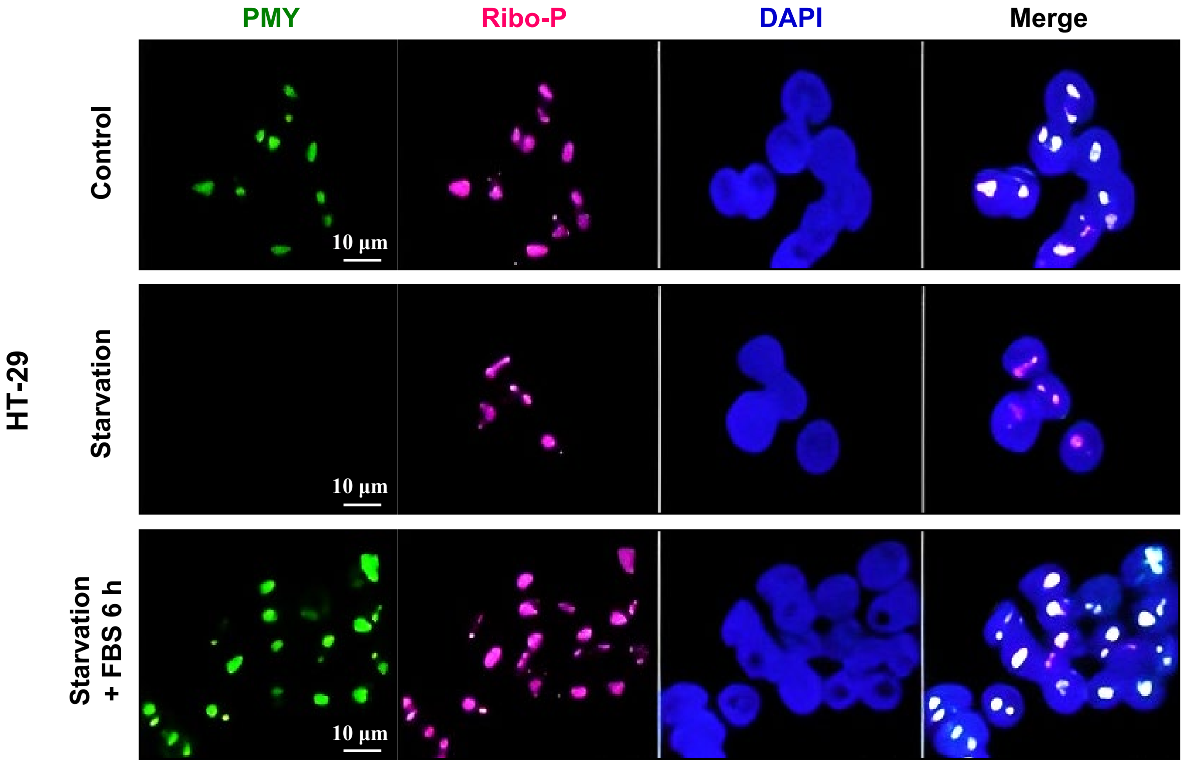

Figure S3. Nuclear translation detected by NRPM in various malignant cancer cell lines with or without serum starvation.

Starvation + FBS 6 h: cells were treated with serum starvation for 48 hours prior to FBS re-supplementation for 6 hours.

Figure S3

G

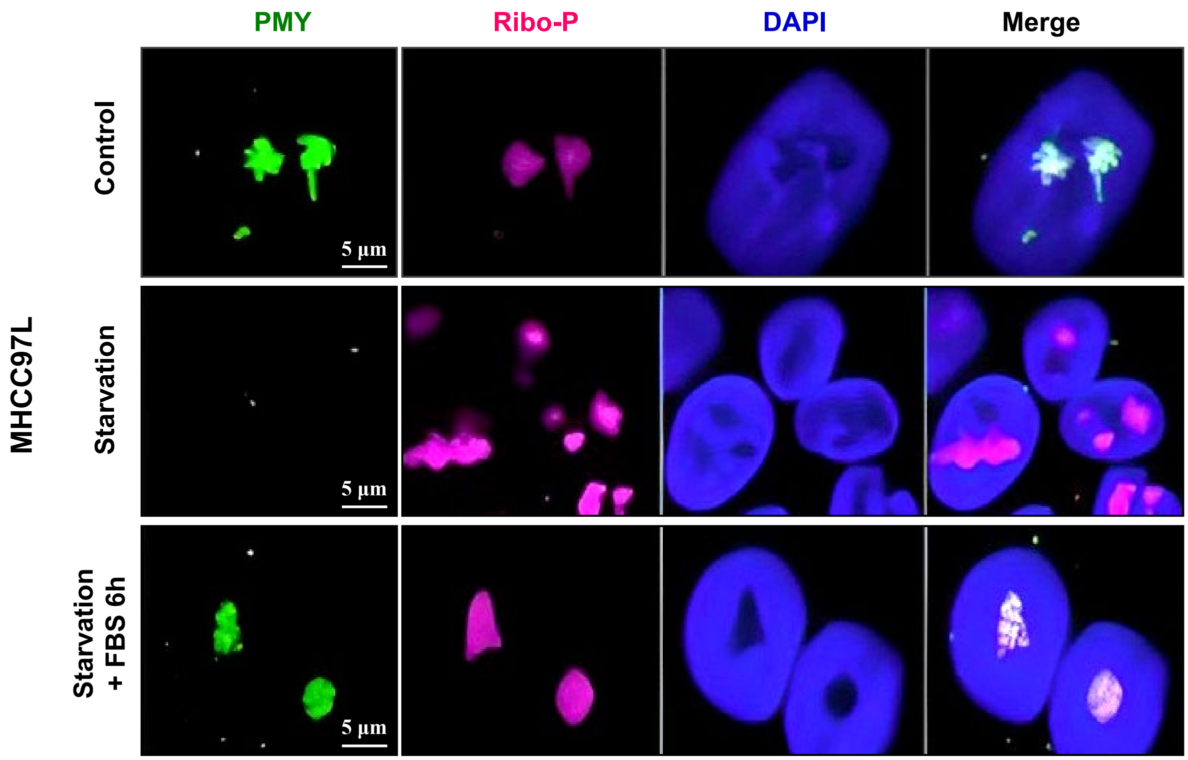

H

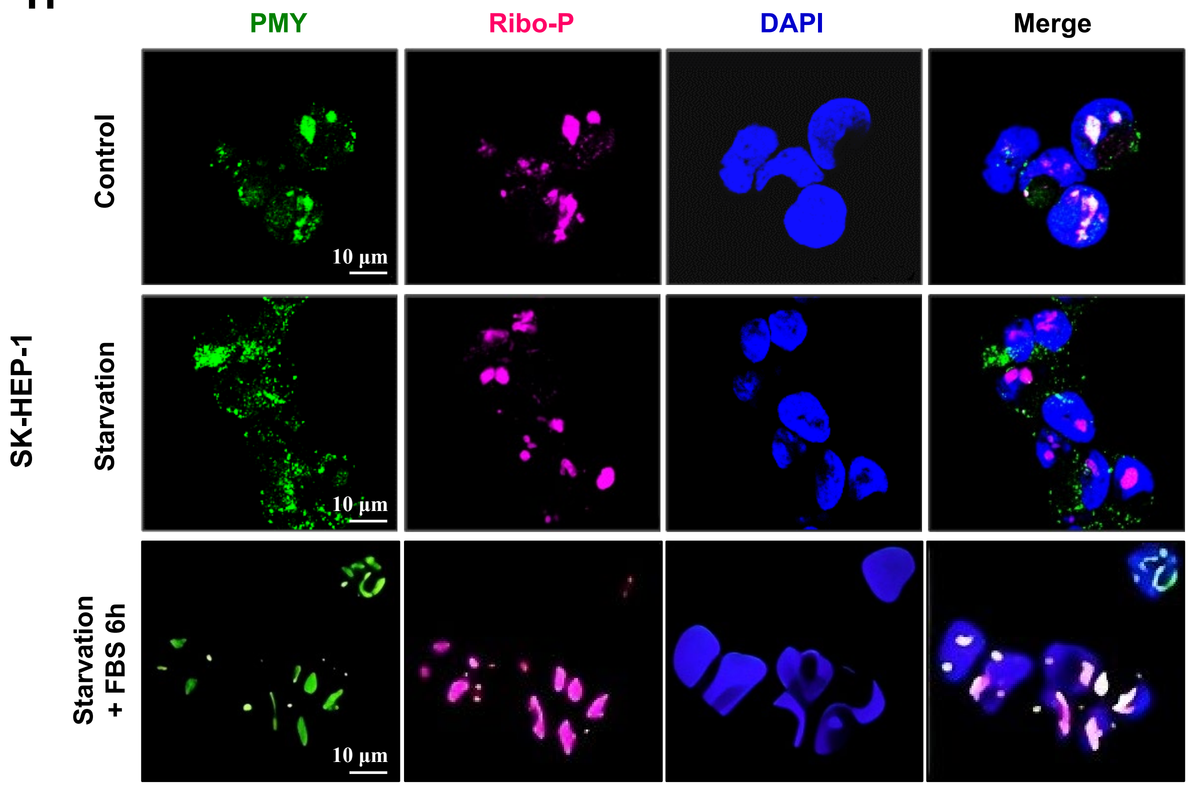

Figure S3. Nuclear translation detected by NRPM in various malignant cancer cell lines with or without serum starvation.

Starvation + FBS 6 h: cells were treated with serum starvation for 48 hours prior to FBS re-supplementation for 6 hours.

Figure S3

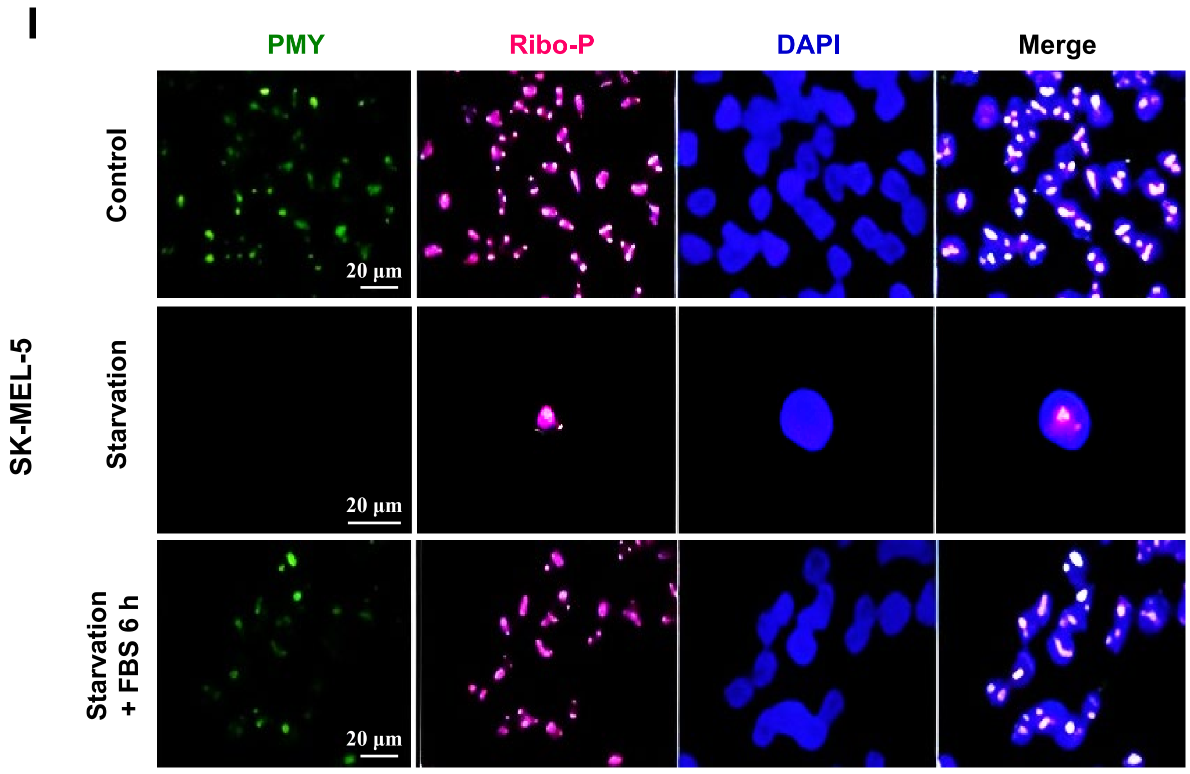

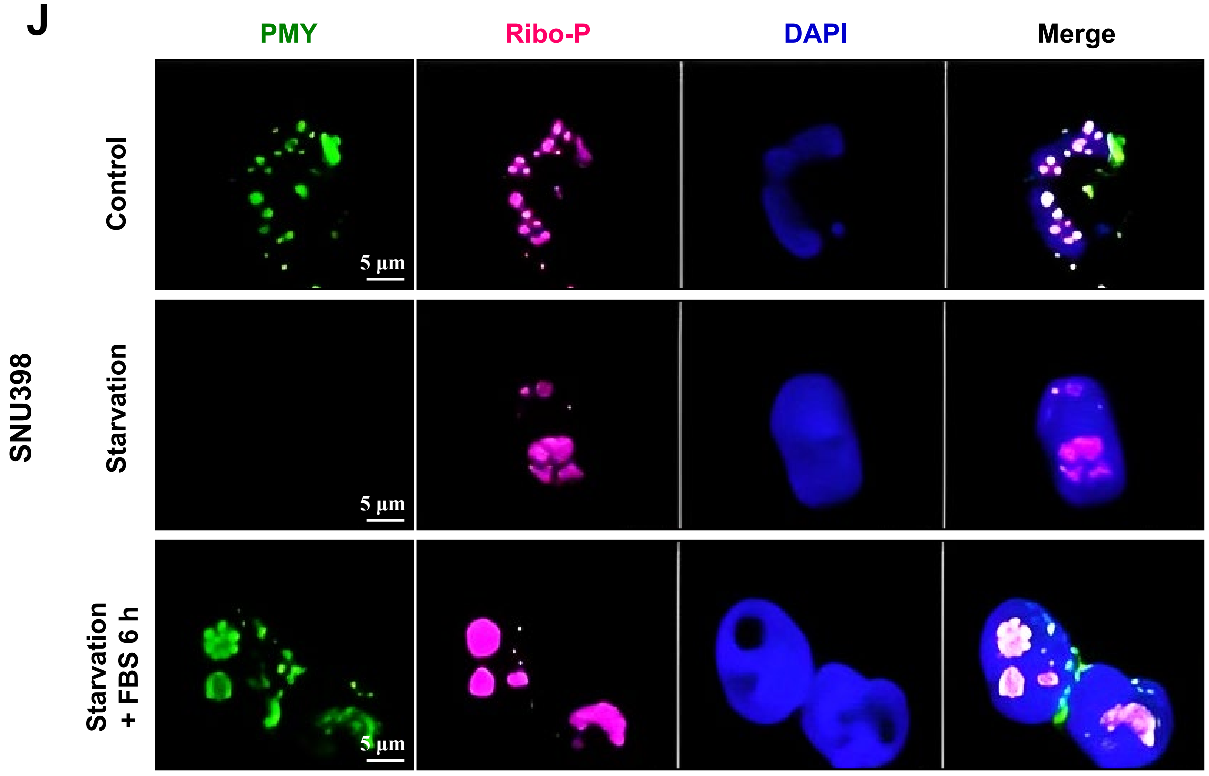

Figure S3. **Nuclear translation detected by NRPM in various malignant cancer cell lines with or without serum starvation.**

Starvation + FBS 6 h: cells were treated with serum starvation for 48 hours prior to FBS re-supplementation for 6 hours.

Figure S3

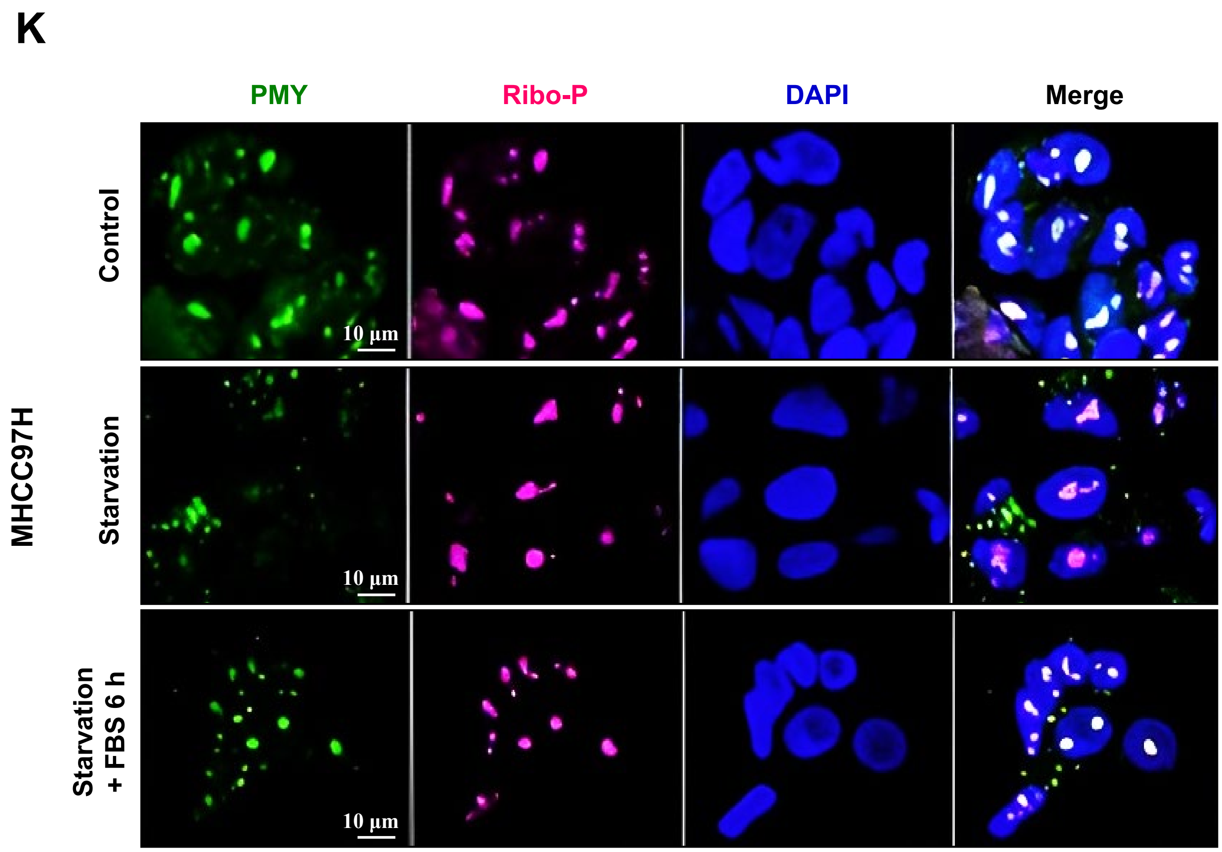

Figure S3. Nuclear translation detected by NRPM in various malignant cancer cell lines with or without serum starvation.

Starvation + FBS 6 h: cells were treated with serum starvation for 48 hours prior to FBS re-supplementation for 6 hours.

Figure S4

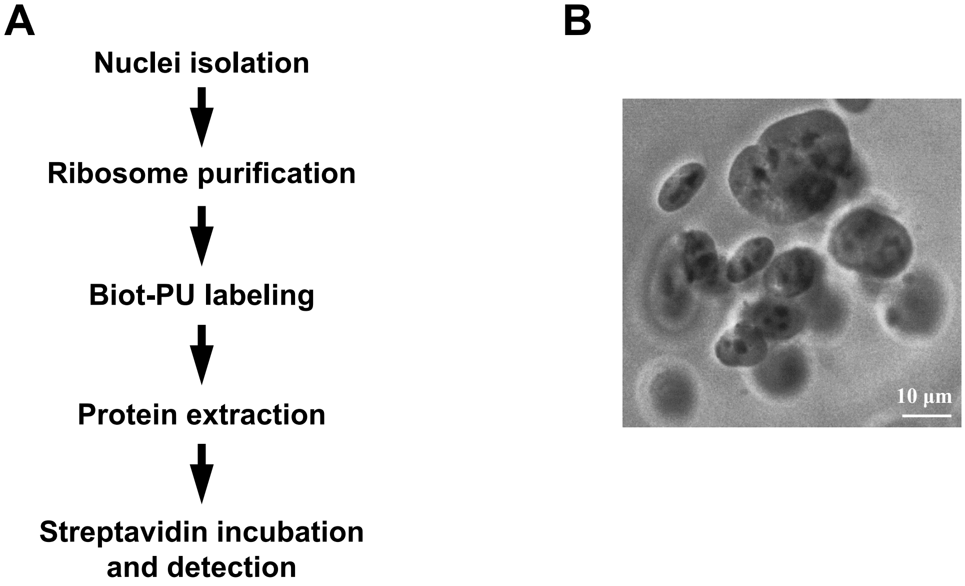

Figure S4. **Modified PUNCH-P for confirming NT.**

(**A**) Schematic outline of the nuclear PUNCH-P analysis. **(B)** Nuclei stained by trypan blue.

Figure S5

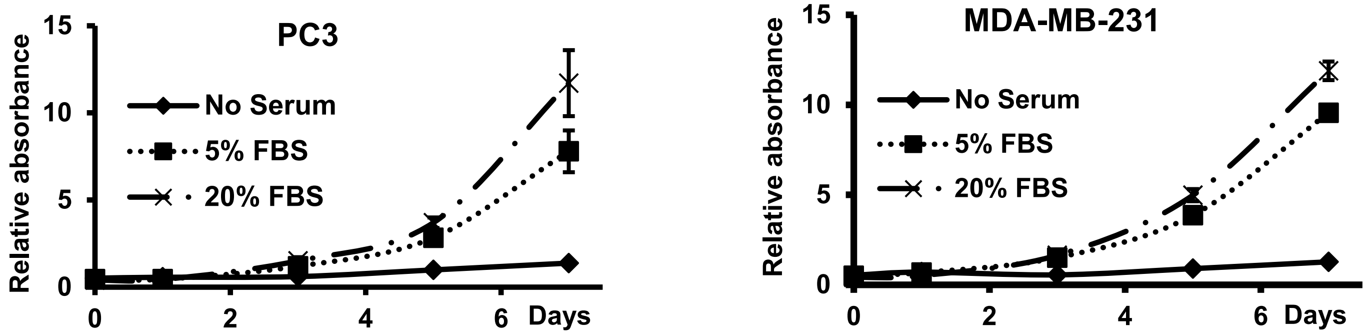

Figure S5. MTS analysis of PC3 and MDA-MB-231 cells cultured with different concentration of fetal bovine serum (FBS) as indicated.

Figure S6

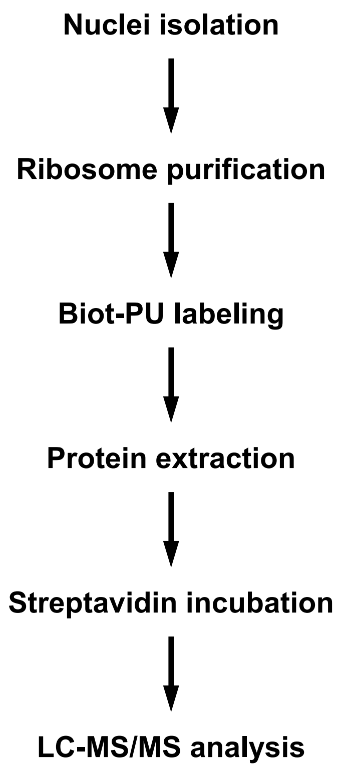

Figure S6. **Schematic outline of the nuclear PUNCH-P analysis.**

Figure S7

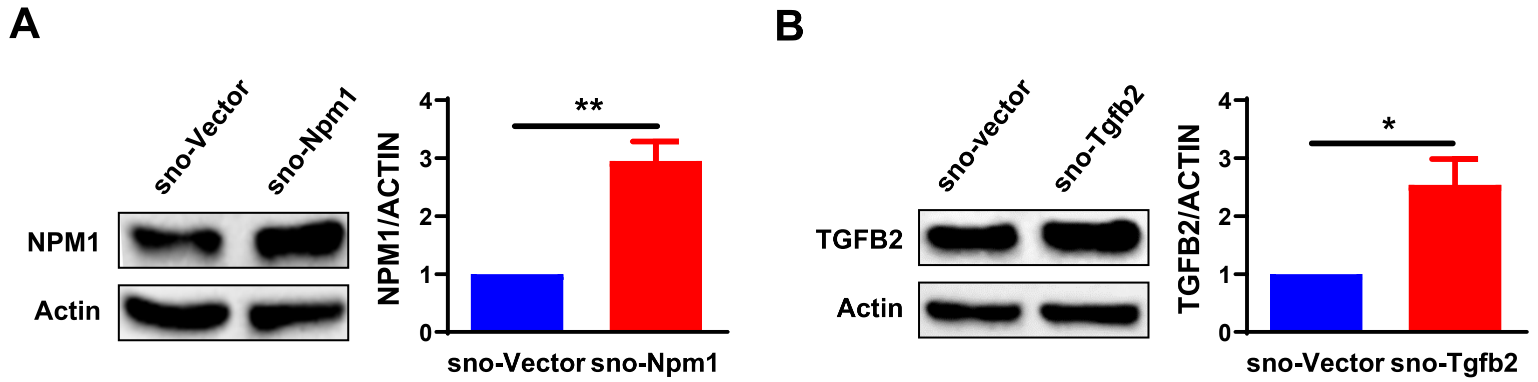

Figure S7. Nuclear accumulation of mNpm1 and mTgfb2 transcripts increases its protein levels in PC3 cells. PC3 cells were transfected with indicated snoVectors, the protein levels of NPM1 **(A)** and TGFB2 **(B)** in whole cells were determined by Western blotting.

Table S1. Oligos used in the study

Genes	Forward primer	Reverse primer
mNpm1 cloning primers	cccaagcttATGGAAGACTCGATGGATATGGA	acgcgtcgacTTAAAGAGATTTCCTCCACTGCC
mTgfb2 cloning primers	ccggaattcATGCACTACTGTGTGCTGAGCA	cggggtaccTTAGCTGCATTTACAAGACTTGACAAT
mNpm1	ATGGAAGACTCGATGGATATGGA	ACCGTTCTTAATGACAACTGGTG
mTgfb2	CTTCGACGTGACAGACGCT	GCAGGGGCAGTGTAAACTTATT
hNpm1	ACGGTCAGTTTAGGGGCTG	CTGTGGAACCTTGCTACCACC
hTgfb2	CAGCACACTCGATATGGACCA	CCTCGGGCTCAGGATAGTCT
hGapdh	ACCACTGACACGTTGGCAGT	ACAGCCTCAAGATCATCAGCAA
hNEAT1	GGAGGGCCGGGAGGGCTAAT	CGGTCAGCCCCGTCGAGCTA

Table S2. Differentially translated proteins between PC3 cells and nuclei as determined by PUNCH-P analysis

Protein	N-1	N-2	N-3	W-1	W-2	W-3	N-T	W-T	N/W	P Value	Reference
TOP2A	44	59	56	0	0	0	159	0	0	0.000	Nat Rev Cancer. 2009;9(5):338-50
CKAP4	44	58	30	0	0	0	132	0	0	0.006	J Clin Invest. 2016;126(7):2689-705
GNL3	35	33	28	0	0	0	96	0	0	0.000	Cancer Lett. 2017;388:220-229.
ATAD3A	32	28	23	0	0	0	83	0	0	0.000	Oncogene. 2016;35(3):333-43.
HDLBP	32	29	26	0	0	0	87	0	0	0.000	Nat Genet. 2014;46(9):964-72.
SCAF1	30	32	24	0	0	0	86	0	0	0.000	Gene. 2018. pii: S0378-1119(18)30533-X.
IGF2BP3	27	31	21	0	0	0	79	0	0	0.001	Oncotarget. 2014;5(16):6832-45.
HNRNPU	27	30	27	0	0	0	84	0	0	0.000	Oncogene. 2018;37(20):2728-2745
COPA	29	27	15	0	0	0	71	0	0	0.006	Cancer Res. 2016; 76(13):3895-903.
PRPF4B	29	20	21	0	0	0	70	0	0	0.001	Cancer Lett. 2013; 335(2):351-60.
RRP1B	26	22	28	0	0	0	76	0	0	0.000	Tumour Biol. 2015; 36(2):615-21.
LUC7L2	21	27	25	0	0	0	73	0	0	0.000	Blood Cancer J. 2013 ; 3(5): e117.
IMMT	25	20	15	0	0	0	60	0	0	0.002	J Cell Physiol. 2016; 231(6):1364-74.
DDX5	21	21	25	0	0	0	67	0	0	0.000	Mol Cell Proteomics. 2012; 11(2):M111.011932.
ATP2A2	25	18	13	0	0	0	56	0	0	0.006	Oncol Rep. 2017; 37(5):2865-2874.
RPN1	21	19	20	0	0	0	60	0	0	0.000	Mol Biol (Mosk). 2011; 45(2):238-48.
RARS	21	19	12	0	0	0	52	0	0	0.003	J Cell Physiol. 2005;204(1):280-5
KTN1	21	17	16	0	0	0	54	0	0	0.000	Neuro Oncol. 2015; 17(10):1365-73.
DDX54	21	16	14	0	0	0	51	0	0	0.001	Front Oncol. 2021 Apr 21;11:650360.
SRP72	17	19	10	0	0	0	46	0	0	0.005	J Mol Cell Biol. 2017; 9(3): 220–230.
RRP1	11	14	18	0	0	0	43	0	0	0.002	PLoS Genet. 2007; 3(11):e214.
ABCF2	15	10	15	0	0	0	40	0	0	0.001	Mol Carcinog. 2017; 56(6):1543-1553.
CLTC	8	15	9	0	0	0	32	0	0	0.008	Genes Chromosomes Cancer. 2014; 53(1):78-89.
PHB	12	14	7	0	0	0	33	0	0	0.006	J Exp Clin Cancer Res. 2018 Feb 7;37(1):21
DHX30	14	9	8	0	0	0	31	0	0	0.005	Cancers (Basel). 2021 Aug 31;13(17):4412.
RBM39	10	11	14	0	0	0	35	0	0	0.001	Cell Oncol (Dordr). 2012; 35(4):293-300.
SRP68	14	10	4	0	0	0	28	0	0	0.033	J Mol Cell Biol. 2017; 9(3): 220–230.
MKI67	14	12	12	0	0	0	38	0	0	0.000	J Clin Pathol. 2013; 66(6):512-6.
PARP1	14	12	13	0	0	0	39	0	0	0.000	Oncotarget. 2015; 6(42):44819-31.
SRRM1	9	13	10	0	0	0	32	0	0	0.001	EBioMedicine. 2020 Jan;51:102547.
GTPBP4	11	12	11	0	0	0	34	0	0	0.000	Biomark Insights. 2017; 12:1177271917730557.
AHNAK	11	12	11	0	0	0	34	0	0	0.000	Oncogene. 2014; 33(38):4675-84.
FLNB	12	10	9	0	0	0	31	0	0	0.000	J Mol Biomark Diagn. 2017; 8(2): 323.
FTSJ3	11	12	9	0	0	0	32	0	0	0.000	RNA Biol. 2020 Apr;17(4):474-486.
SEC61B	7	11	10	0	0	0	28	0	0	0.001	Clin Chim Acta. 2011;412(11-12):887-93
EPRS	11	6	11	0	0	0	28	0	0	0.005	Oncotarget. 2016; 7(43):69592-69605
ATP1A1	10	7	10	0	0	0	27	0	0	0.001	Oncotarget. 2016; 7(51):85244-85258
DDX18	8	5	10	0	0	0	23	0	0	0.006	Oncogene. 2015; 34(29):3871-80
MYBBP1A	9	7	5	0	0	0	21	0	0	0.004	Cancer Lett. 2015; 358(2):191-199
SEC63	9	9	7	0	0	0	25	0	0	0.000	Scand J Gastroenterol. 2013; 48(3):344-51
STAU1	7	9	5	0	0	0	21	0	0	0.004	Cell Death Dis. 2017; 8(6):e2837
DDX3X	9	9	3	0	0	0	21	0	0	0.025	Cancer Res. 2016, 76(21): 6340-50.
SRSF11	9	8	9	0	0	0	26	0	0	0.000	Cancer Res. 2013, 73(9): 2817-28.
PTBP1	5	9	9	0	0	0	23	0	0	0.005	Oncogenesis. 2014; 3: e84.
PPAN	7	9	7	0	0	0	23	0	0	0.000	Biochim Biophys Acta Mol Cell Res.2019;(5):882-95.
MARS	5	9	7	0	0	0	21	0	0	0.004	BMC Cancer. 2017 Jul 5;17(1):467
HIST1H1E	9	6	3	0	0	0	18	0	0	0.026	Bioinformatics. 2015;31(22):3561-8
STAU2	8	7	7	0	0	0	22	0	0	0.000	Oncotarget. 2018; 9(35): 24140-24154.
LUC7L3	8	7	8	0	0	0	23	0	0	0.000	Clin Cancer Res. 2014;20(15):4129-40

DDX52	8	8	8	0	0	0	24	0	0	0.000	Bioengineered. 2021; 12(1):3539-3549.
TTC37	4	7	8	0	0	0	19	0	0	0.006	
EIF2AK2	5	5	7	0	0	0	17	0	0	0.001	Mol Med Rep. 2017;16(5): 7585-7590.
ITGB1	7	6	5	0	0	0	18	0	0	0.000	Sci Rep. 2018;8(1):2338
QSOX1	7	6	4	0	0	0	17	0	0	0.003	Oncotarget. 2015; 6(21): 18418-28.
HSPA1A	4	4	7	0	0	0	15	0	0	0.007	Cancer Lett. 2012;317(2):157-64
LRPPRC	2	5	7	0	0	0	14	0	0	0.033	Cancer. 2014;120(8):1228-36
NOMO1	5	2	7	0	0	0	14	0	0	0.033	Oncotarget. 2017;8(15):24429-24436
NUP205	6	2	6	0	0	0	14	0	0	0.025	Epigenetics Chromatin. 2016; 9-54.
PTPLAD1	6	5	4	0	0	0	15	0	0	0.001	Int J Cancer. 2012;131(11):2499-508
REXO4	6	3	4	0	0	0	13	0	0	0.008	Cancer Genet. 2016; 209(6): 251-7.
PGAM5	4	2	6	0	0	0	12	0	0	0.026	Nat Commun. 2015;6:8371.
ZC3HAV1	4	6	3	0	0	0	13	0	0	0.008	Nat Commun. 2014;5:5362
EEF1D	2	6	4	0	0	0	12	0	0	0.026	J Exp Clin Cancer Res. 2018; 37(1):50.
DDX46	5	6	2	0	0	0	13	0	0	0.023	Oncol Res. 2017; 25(3): 417-425.
CDKN2A	4	6	2	0	0	0	12	0	0	0.026	Clin Cancer Res. 2014;20(6):1676-86.
DKK1	6	5	2	0	0	0	13	0	0	0.023	Oncotarget. 2015; 6(32): 32410-25.
SMARCA4	6	4	2	0	0	0	12	0	0	0.026	Cancer Res. 2014; 74(22): 6486-6498.
EIF3E	5	4	6	0	0	0	15	0	0	0.001	Nature. 2018;561(7724):556-560
HNRNPK	5	6	5	0	0	0	16	0	0	0.000	Oncotarget. 2017; 8(61): 103364-103374.
EIF3A	4	6	2	0	0	0	12	0	0	0.026	Cell Oncol (Dordr). 2014 , 37(4): 253-67.
RBM34	2	5	4	0	0	0	11	0	0	0.014	J Clin Transl Hepatol.2023, 28;11(2):369-381.
LGALS3BP	2	5	4	0	0	0	11	0	0	0.014	Int J Oncol. 2016;49(1):265-75
EFTUD2	4	5	3	0	0	0	12	0	0	0.002	Cancer Med. 2015;4(2):268-77
TGFB2	2	4	5	0	0	0	11	0	0	0.014	Nat Cell Biol. 2013;15(11):1351-6
SPTAN1	4	5	3	0	0	0	12	0	0	0.002	Oncotarget. 2016; 8(35): 58098-58107.
LDHA	2	5	3	0	0	0	10	0	0	0.019	Tumour Biol. 2015 ; 36(10): 8093-100.
SMC2	4	2	2	0	0	0	8	0	0	0.016	APMIS. 2014; 122(6): 499-504.
MBOAT7	4	3	2	0	0	0	9	0	0	0.007	Sci Rep. 2017; 7(1): 4492.
TOP2B	3	1	4	0	0	0	8	0	0	0.039	Nat Genet. 2010; 42(8): 668-75.
EIF3H	2	4	2	0	0	0	8	0	0	0.016	Carcinogenesis. 2014; 35(3): 670-82.
EIF3C	4	3	4	0	0	0	11	0	0	0.000	Med Sci Monit. 2017; 23: 4182-4191.
CTNNB1	2	4	4	0	0	0	10	0	0	0.007	Carcinogenesis. 2012;33(3):501-8
COPG	3	3	1	0	0	0	7	0	0	0.025	J Proteome Res. 2008; 7(3): 1138-50.
PTGES	3	2	2	0	0	0	7	0	0	0.002	Oncotarget. 2015;6(37):39941-59
LRRC59	2	3	2	0	0	0	7	0	0	0.002	Onco Targets Ther. 2020 Jul 3;13:6453-6463.
SQRDL	2	1	2	0	0	0	5	0	0	0.007	Oncotarget. 2017;8(61):104508-104524
USP42	1	1	1	0	0	0	3	0	0	0.000	Oncogene. 2017;36(42):5819-5828
GCN1L1	22	23	23	1	0	0	68	1	68.0	0.000	
LAMB3	16	20	13	0	0	1	49	1	49.0	0.001	Cancer Res. 2016, 76(19): 5777-87.
TFRC	22	30	26	2	0	0	78	2	39.0	0.000	Ann Surg Oncol. 2006;13(7):961-6
PRKDC	275	304	233	13	4	7	812	24	33.8	0.000	Cancer Discov. 2014;4(5):592-605
PDIA3	6	11	9	1	0	0	26	1	26.0	0.005	Genet Mol Res. 2015; 14(2):6960-7.
CYR61	12	8	5	0	1	0	25	1	25.0	0.018	J Biol Chem, 2016, 291(53): 27433
STT3A	20	13	13	2	0	0	46	2	23.0	0.004	aryngoscope. 2011;121(5):983-9
HP1BP3	35	42	35	3	0	2	112	5	22.4	0.000	Mol Cell Proteomics.2014, 13(12):3236-49
C3	23	23	18	0	0	3	64	3	21.3	0.000	Cytokine. 2014; 66(1):60-8
DDX24	6	8	7	0	0	1	21	1	21.0	0.001	Oncogene. 2016;35(4):528-36
VCL	11	8	9	0	2	0	28	2	14.0	0.001	Am J Surg Pathol. 2014; 38(6): 858–863.
HSP90B1	15	19	20	0	4	0	54	4	13.5	0.001	PLoS One. 2012; 7(2): e30992.
HNRNPM	7	8	11	0	0	2	26	2	13.0	0.004	Cancer Res. 2018;78(20):5780-92

MTDH	5	3	5	0	1	0	13	1	13.0	0.006	Int J Clin Exp Med. 2015; 8(4): 4795-807.
CSE1L	5	4	4	1	0	0	13	1	13.0	0.001	Am J Surg. 2013; 206(3): 418-27.
PCBP1	5	5	2	1	0	0	12	1	12.0	0.025	Sci Rep. 2017; 7(1): 15488.
ACTN1	14	11	7	1	2	0	32	3	10.7	0.010	PLoS One. 2015; 10(4): e0120616.
SND1	23	26	13	3	3	0	62	6	10.3	0.010	Cancer Res. 2015; 75(7): 1275-86.
HNRNPR	2	3	4	0	0	1	9	1	9.0	0.016	Aging (Albany NY). 2019, 16;11(18):7473-7491.
RSL1D1	33	36	42	4	3	6	111	13	8.5	0.000	Oncol Lett. 2016; 11(4): 2839-2844.
SRSF6	5	5	7	0	0	2	17	2	8.5	0.006	Gut. 2017. pii: gutjnl-2017-314983.
HSPA5	24	27	22	0	4	5	73	9	8.1	0.001	Ann Surg Oncol. 2015; 22(3): 889-98.
EEF2	15	13	11	2	0	3	39	5	7.8	0.001	Int J Oncol. 2014; 44(5):1461-9.
LCN2	9	10	9	2	2	0	28	4	7.0	0.000	Elife. 2017;6. pii: e23242
ATP5A1	12	12	9	2	0	3	33	5	6.6	0.002	J Neurooncol. 2016; 126(3): 405-13.
EIF4G1	17	14	15	4	3	0	46	7	6.6	0.001	PNAS. 2012; 109(46): 18767-72.
SEC61A1	30	24	20	5	7	0	74	12	6.2	0.004	J Cell Biochem. 2019 Apr;120(4):6145-6153.
THBS1	62	65	52	13	9	10	179	32	5.6	0.000	Anticancer Res. 2015; 35(4): 2071-6.
HSPA6	9	12	9	3	3	0	30	6	5.0	0.005	Int J Med Sci. 2015;12(3):256-63
CXCL1	18	18	11	2	3	5	47	10	4.7	0.008	Cancer Lett. 2015; 359(2): 335-43.
NPM1	21	20	25	5	6	4	66	15	4.4	0.000	Oncotarget. 2015; 6(25): 21443-51.
KRT7	24	18	15	4	3	7	57	14	4.1	0.008	Oncogene. 2016; 35(37): 4927-36.
AKAP12	22	28	22	7	5	6	72	18	4.0	0.001	Int J Oncol. 2018 ; 52(4): 1305-1316.
SNRNP70	4	7	5	1	3	0	16	4	4.0	0.033	Anal Cell Pathol. 2011; 34(3): 159-68.
RRBP1	66	48	54	15	20	9	168	44	3.8	0.003	Cancer Manag Res. 2018; 10: 1243-1249.
FLNA	49	44	59	19	11	13	152	43	3.5	0.002	Biomark Med. 2016; 10(7): 711-9.
ILF3	16	21	21	8	5	4	58	17	3.4	0.003	Exp Ther Med. 2010; 1(1): 187-192.
DHX9	11	10	8	7	2	0	29	9	3.2	0.042	Am J Transl Res. 2017; 9(2): 674-682.
HSP90AB1	26	22	31	6	9	11	79	26	3.0	0.004	Oncogene. 2018. PMID: 30305727
ABCF1	5	4	3	2	1	1	12	4	3.0	0.016	Curr Protein Pept Sci. 2015; 16(4): 301-9.
RPL6	108	127	99	35	36	50	334	121	2.8	0.002	PLoS One. 2011; 6(10): e26401.
RPL37	14	18	23	8	6	6	55	20	2.8	0.012	Biochim Biophys Acta. 1994; 1218: 425-8
ACTB	68	51	39	15	19	25	158	59	2.7	0.021	Clin Chim Acta. 2013; 417: 39-44
MYADM	16	19	15	10	8	1	50	19	2.6	0.026	Life Sci. 2007; 80(5): 420-9.
SRPK1	21	16	17	8	7	6	54	21	2.6	0.003	Onco Targets Ther. 2018; 11: 1671-1681.
RPS11	17	18	10	7	5	6	45	18	2.5	0.025	PLoS One. 2015; 10(10): e0141334.
RPL29	32	30	23	11	12	12	85	35	2.4	0.004	Dis Model Mech. 2013;6(1):115-24
EEF1A1	25	25	14	8	7	13	64	28	2.3	0.043	Transl Oncol. 2018; 11(1): 125-131
MYH9	15	19	14	8	9	4	48	21	2.3	0.014	Gene. 2018; 664: 152-167
RPL19	45	37	38	15	18	22	120	55	2.2	0.003	PLoS One.2011; 6(7): e22672.
RPL8	46	48	43	17	15	31	137	63	2.2	0.009	Cancer Res. 2007; 67(8): 3555-9.
HSPA8	42	47	49	22	26	17	138	65	2.1	0.002	Onco Targets Ther. 2016;9:2169-79
RPL7A	81	82	65	41	37	35	228	113	2.0	0.003	Int J Oncol. 2000;16(4):757-62
RPL10	54	52	45	26	27	22	151	75	2.0	0.001	Oncotarget. 2017; 8(21): 35009-35018.
HSP90AA1	53	51	59	28	24	29	163	81	2.0	0.001	Onco Targets Ther. 2018; 11: 3013-3023.
RPS25	27	25	25	13	12	15	77	40	1.9	0.000	Oncogene. 2013;32(22):2782-91
RPL36	29	31	26	12	12	21	86	45	1.9	0.015	Pathol Int. 2011;61(11):638-44
RPL4	170	183	154	95	82	93	507	270	1.9	0.001	Oncotarget. 2016;7(13):16217-26
RPL15	73	75	52	33	33	45	200	111	1.8	0.024	DNA Cell Biol. 2011;30(9):671-9
RPL34	54	55	56	32	29	34	165	95	1.7	0.000	Gene.2016 ; 576(1Pt3): 421-8.
RPL32	25	30	25	17	14	18	80	49	1.6	0.007	Mol Ther Nucleic Acids. 2020, 4;21:75-85.
UBA52	18	22	21	12	13	13	61	38	1.6	0.004	Cancer Res. 1991;51(24):6677-85
RPL3	67	73	59	47	41	39	199	127	1.6	0.007	Sci Rep. 2016; 6: 38369.
RPL23	2	0	0	7	12	6	2	25	0.08	0.018	Ann Hematol. 2012; 91(10):1547-54.

KRT6B	6	9	53	98	96	102	68	296	0.23	0.008	Oncol Lett. 2017; 14(4): 4633–4640.
KRT17	16	12	5	52	42	49	33	143	0.23	0.001	Gastric Cancer. 2017; 20(6):948-959.
RPS19	4	2	3	17	14	7	9	38	0.24	0.033	PLoS One. 2012; 7(4):e33619.
PABPC1	2	4	0	11	8	6	6	25	0.24	0.027	Int J Clin Exp Pathol. 2015; 8:3794-802.
FLG2	2	2	2	7	7	6	6	20	0.30	0.000	
DSG1	7	9	4	22	25	19	20	66	0.30	0.002	Pathol Res Pract. 2015; 211(3):208-13.
DSP	23	28	19	73	68	63	70	204	0.34	0.000	J Histochem Cytochem. 2017; 65:139-151
KRT6A	5	6	19	24	28	32	30	84	0.36	0.024	Pediatr Dermatol. 2016;33(3):337-42.
JUP	10	10	12	37	26	18	32	81	0.40	0.042	Toxicol Sci. 2013; 134(2):258-70.
NCL	13	15	8	33	20	28	36	81	0.44	0.026	J Exp Med. 2013; 210(5):951-68.
KPRP	4	3	5	11	9	7	12	27	0.44	0.018	
KRT16	46	46	46	102	91	92	138	285	0.48	0.000	PNAS. 2013; 110(48):19537-42.
RPS16	4	4	5	8	8	7	13	23	0.57	0.002	Sci Rep. 2017; 7(1):8365.
KRT5	44	47	41	66	76	89	132	231	0.57	0.009	Nat Cell Biol. 2014; 16(4):345-56.
KRT14	118	122	95	178	181	192	335	551	0.61	0.002	Nat Commun. 2016 Jun 20;7:11914

N: nuclei; W: whole cell; N-T: Nuclei Total; W-T: Whole cell Total; N/W: nuclei/whole cell

Proteins in RED have oncogenic activity. Proteins in BLUE display anti-tumor activity.

Proteins in BLACK mean their role in cancer have not yet determined.

Table S3. GO analysis on proteins that are preferentially translated in the nucleus

Term	Count	%	P value	Genes	Fold enrichment	FDR
Translation	28	1	1.27E-14	ABCF1, RPL19, RPL15, RPL36, RPL37, EIF3A, RPL32, EIF3H, RPL6, RPL34, EIF3E, NPM1, RPL8, RPL3, RPL10, RPL7A, THBS1, MARS, PTBP1, EPRS, ILF3, RPL29, EIF4G1, RARS, RPS11, EIF2AK2, LRPPRC, UBA52	6.38	2.26E-11
Peptide biosynthetic process	28	0.094	2.24E-14	ABCF1, RPL19, RPL15, RPL36, RPL37, EIF3A, RPL32, EIF3H, RPL6, RPL34, EIF3E, NPM1, RPL8, RPL3, RPL10, RPL7A, THBS1, MARS, PTBP1, EPRS, ILF3, RPL29, EIF4G1, RARS, RPS11, EIF2AK2, LRPPRC, UBA52	6.23	3.97E-11
Amide biosynthetic process	29	0.097	3.85E-14	ABCF1, RPL19, RPL15, RPL36, RPL37, EIF3A, RPL32, EIF3H, RPL6, RPL34, EIF3E, NPM1, RPL8, RPL3, RPL10, RPL7A, THBS1, CYR61, MARS, PTBP1, EPRS, ILF3, RPL29, EIF4G1, RARS, RPS11, EIF2AK2, LRPPRC, UBA52	5.81	6.82E-11
Peptide metabolic process	28	0.094	2.75E-14	ABCF1, RPL19, RPL15, RPL36, RPL37, EIF3A, RPL32, EIF3H,	5.10	4.88E-11

				RPL6, RPL34, EIF3E, NPM1, RPL8, RPL3, RPL10, RPL7A, THBS1, MARS, PTBP1, EPRS, ILF3, RPL29, EIF4G1, RARS, RPS11, EIF2AK2, LRPPRC, UBA52		
Cellular amide metabolic process	20	0.097	2.09E-11	ABCF1, RPL19, RPL15, RPL36, RPL37, EIF3A, RPL32, EIF3H, RPL6, RPL34, EIF3E, NPM1, RPL8, RPL3, RPL10, RPL7A, THBS1, CYR61, MARS, PTBP1, EPRS, ILF3, RPL29, EIF4G1, RARS, RPS11, EIF2AK2, LRPPRC, UBA52	4.48	3.70E-08
Organonitrogen compound biosynthetic process	32	0.107	9.56E-11	ABCF1, RPL19, RPL15, AKAP12, RPL36, RPL37, TGFB2, EIF3A, RPL32, EIF3H, RPL6, RPL34, EIF3E, NPM1, RPL8, RPL3, RPL10, RPL7A, THBS1, CYR61, MARS, PTBP1, EPRS, ILF3, RPL29, EIF4G1, RARS, ATP5A1, RPS11, EIF2AK2, LRPPRC, UBA52	3.79	1.69E-08
Gene expression	67	0.225	1.64E-10	ABCF1, RPL19, HP1BP3, RPL15, TGFB2, CTNNB1, CDKN2A, RRP1B, SND1, PCBP1, RPL10, SRRM1, DHX30, FTSJ3, AHNAK, LUC7L3, CYR61, GNL3, HIST1H1E, RRP1, PTBP1, MYH9,	1.99	2.91E-07

				MYADM, SRPK1, HNRNPU, EIF4G1, RSL1D1, RARS, RBM39, RPS11, EIF2AK2, MYBBP1A, UBA52, LRPPRC, SMARCA4, MTDH, PRKDC, RPL36, RPL37, HSPA1A, HNRNPM, EIF3A, HNRNPK, DDX46, RPL32, RPL6, EIF3H, RPL34, EIF3E, RPL8, NPM1, RPL3, SNRNP70, RPL7A, THBS1, HSPA8, MARS, DHX9, PHB, EPRS, ILF3, DDX5, RPL29, DKK1, SRSF6, DDX54, DDX52		
RNA processing	21	0.070	1.46E-07	DHX9, RRP1, PTBP1, HSPA1A, DDX5, RSL1D1, HNRNPM, HNRNPK, DDX46, RRP1B, SRSF6, SRRM1, SNRNP70, RPL7A, RBM39, DDX54, DHX30, FTSJ3, DDX52, LUC7L3, HSPA8	4.07	2.59E-04
Ribonucleoprotein complex biogenesis	16	0.054	2.47E-07	RRP1, RSL1D1, PPAN, EIF3A, RRP1B, EIF3H, SRSF6, RPL6, EIF3E, RPL34, RPL7A, DHX30, DDX52, LUC7L3, FTSJ3, GNL3	5.35	4.37E-04
Cytoplasmic translation	7	0.023	2.26E-06	EIF3A, RPL6, RPL15, RPL8, PTBP1, RPL36, RPL29	17.96	0.004

Table S4. KEGG analysis on proteins that are preferentially translated in the nucleus

Term	Count	%	P value	Genes	Fold enrichment	FDR
Ribosome	17	0.057	1.16E-10	RPL19, RPL15, RPL36, RPL37, RPL29, RPS25, RPL32, RPL6, RPL34, RPL8, RPL3, RPL10, RPL7A, RPL4, RPS11, UBA52	8.21	1.34E-07
Protein processing in endoplasmic reticulum	14	0.047	8.74E-08	HSP90AB1, HSP90AA1, PDIA3, RRBP1, CKAP4, SEC63, HSP90B1, STT3A, SEC61B, RPN1, HSPA5, EIF2AK2, HSPA8, SEC61A1	6.80	1.02E-04
Protein export	6	0.020	8.43E-06	SEC61B, SRP68, SRP72, HSPA5, SEC61A1, SEC63	20.29	0.001
Spliceosome	10	0.033	3.65E-05	HNRNPM, HNRNPK, DDX46, SRSF6, EFTUD2, PCBP1, SNRNP70, DDX5, HSPA8, HNRNPU	5.97	0.04
Phagosome	7	0.023	0.011	ACTB, SEC61B, TFRC, C3, THBS1, ITGB1, SEC61A1	3.64	12.67
Focal adhesion	8	0.027	0.013	ACTB, LAMB3, ACTN1, THBS1, FLNB, ITGB1, CTNNB1, VCL	3.11	14.48
RNA transport	7	0.023	0.015	EIF4G1, EEF1A1, EIF3A, EIF3H, NUP205, EIF3E, SRRM1	3.46	15.68
Bacterial invasion of epithelial cells	5	0.017	0.016	ACTB, CLTC, ITGB1, CTNNB1, VCL	5.07	17.15
Proteoglycans in cancer	7	0.023	0.036	ACTB, DDX5, THBS1, FLNB, ITGB1, TGFB2, CTNNB1	2.81	34.71
Viral carcinogenesis	7	0.023	0.036	CDKN2A, HNRNPK, DDX3X, C3, SND1, ACTN1, EIF2AK2	2.81	34.71
